# A meta-analysis of event-related potential correlates of recognition ﻿memory

**DOI:** 10.3758/s13423-023-02309-y

**Published:** 2023-07-11

**Authors:** Simon Kwon, Michael D. Rugg, Ronny Wiegand, Tim Curran, Alexa M. Morcom

**Affiliations:** 1 Department of Psychology, University of Edinburgh, Edinburgh, UK; 2 Department of Psychology, University of Cambridge, Cambridge, UK; 3 Department of Psychiatry, Brigham and Women’s Hospital, Boston, MA USA; 4 Center for Vital Longevity and School of Behavioral and Brain Sciences, University of Texas at Dallas, Dallas, TX USA; 5 School of Psychology, University of East Anglia, Norwich, UK; 6 Department of Psychology and Neuroscience, University of Colorado Boulder, Boulder, CO USA; 7 School of Psychology, University of Sussex, Brighton, UK

**Keywords:** Recollection, Familiarity, Recognition memory, Event-related potentials, Meta-analysis

## Abstract

A longstanding question in memory research is whether recognition is supported by more than one mnemonic process. Dual-process models distinguish recollection of episodic detail from familiarity, while single-process models explain recognition in terms of one process that varies in strength. Dual process models have drawn support from findings that recollection and familiarity elicit distinct electroencephalographic event-related potentials (ERPs): a mid-frontal ERP effect that occurs at around 300–500 ms post-stimulus onset and is often larger for familiarity than recollection contrasts, and a parietal ERP effect that occurs at around 500–800 ms and is larger for recollection than familiarity contrasts. We sought to adjudicate between dual- and single-process models by investigating whether the dissociation between these two ERP effects is reliable over studies. We extracted effect sizes from 41 experiments that had used Remember-Know, source memory, and associative memory paradigms (1,000 participants). Meta-analysis revealed a strong interaction between ERP effect and mnemonic process of the form predicted by dual-process models. Although neither ERP effect was significantly process-selective taken alone, a moderator analysis revealed a larger mid-frontal effect for familiarity than recollection contrasts in studies using the Remember-Know paradigm. Mega-analysis of raw data from six studies further showed significant process-selectivity for both mid-frontal and parietal ERPs in the predicted time windows. On balance, the findings favor dual- over single-process theories of recognition memory, but point to a need to promote sharing of raw data.

**Supplementary Information:**

The online version contains supplementary material available at 10.3758/s13423-023-02309-y.

## Introduction

The ability to recognize previously encountered information, or recognition memory, has been the subject of an enduring theoretical debate. According to dual-process accounts, recognition memory is based on two kinds of mnemonic evidence, supported by distinct neural substrates, that explain qualitatively different mnemonic experiences: recollection and familiarity (Atkinson & Juola, 1974; Mandler, 1980; Rugg & Curran, 2007; Yonelinas et al., 2002). Recollection refers to a detailed and often vivid experience of “reliving the past” that entails the recovery of episodic information such as where and when the original encounter occurred (Tulving, 1983). In contrast, familiarity refers to the undifferentiated experience of “just knowing” that something was previously encountered, without retrieval of contextual information. Despite these distinct subjective experiences, single-process accounts propose that only one kind of mnemonic evidence is used to support recognition memory, relying on a single neural substrate (Donaldson, 1996; Dunn, 2004; Squire et al., 2007). On this view, recollection reflects a stronger and familiarity a weaker memory signal. These two competing theoretical views have been difficult to resolve with only behavioral methods because different dual- and single-process models differ in their specific predictions, and can both explain the same behavioral data (e.g., Parks & Yonelinas, 2007; Wixted, 2007; Yonelinas & Parks, 2007; but see Wixted & Mickes, 2010).

Converging evidence has therefore been sought from dissociations between the neural correlates of recollection and familiarity. Leading dual-process theories assume that these two processes depend on neural circuits involving hippocampus and perirhinal cortex, respectively (Aggleton & Brown, 1999; see Yonelinas et al., 2010). This assumption is partially supported by data from patients with selective damage to these circuits (Huppert & Piercy, 1978; see Montaldi & Mayes, 2010, for a review). In patients with selective hippocampal damage, recollection-based memory judgments tend to be impaired compared to healthy controls while familiarity-based judgments are preserved, consistent with a dual-process account (Yonelinas et al., 2002). A few studies have nevertheless reported similar patients who have impairments in both recollection and familiarity, as predicted by a single-process view (Manns et al., 2003; Wixted & Squire, 2004). Further consistent with a reduction in memory strength rather than a difference in the processes involved, comparable behavioral receiver operating characteristic (ROC) curves have been reported for patients with hippocampal damage and controls when memory strength is matched, suggesting that apparent qualitative ROC differences may simply reflect differences in memory strength (Wais et al., 2006). Moreover, lesion studies in humans offer limited evidence for the predicted double dissociation between recollection and familiarity because patients with a selective deficit in familiarity are very rare ( Bowles et al., 2007; Edelstyn et al., 2016). Data from animal models of recognition memory are similarly controversial. While hippocampal lesions in rats alter their ROC curves as would be expected if the rats now rely only on a strength-based familiarity process (Fortin et al., 2004; Sauvage et al., 2008), this interpretation of the ROC data is also contested (Eichenbaum et al., 2010; Wixted & Squire, 2008).

### Event-related potential (ERP) correlates of familiarity and recollection

Given the limitations of behavioral and lesion evidence, dual-process theory has placed particular emphasis on evidence stemming from neuroimaging methods such as electroencephalographic event-related potentials (ERPs). Two retrieval-related ERP effects – the mid-frontal and the parietal old/new effects – have been reported to distinguish familiarity from recollection (Friedman & Johnson, 2000; Mecklinger, 2000; Rugg & Curran, 2007). These ERP effects have been demonstrated within experiments to be temporally and topographically distinct, providing evidence that they reflect different mnemonic processes (e.g., Curran, 2000; Curran & Hancock, 2007; Rugg et al., 1998a; Wilding & Rugg, 1996; Woodruff et al., 2006). However, it is unknown whether this finding generalizes over experiments.

In recognition memory tasks, ERPs are typically more positive-going for correctly identified studied or “old” items compared to those correctly rejected as unstudied or “new” (Sanquist et al., 1980; Warren, 1980). The mid-frontal old/new effect typically onsets earlier, around 300 ms post-stimulus, offsets at around 500 ms, and tends to be maximal over the frontal scalp (Düzel et al., 1997; Rugg et al., 1998a). This effect is also referred to as the FN400, reflecting its modulation of frontal negativity. The parietal ERP old/new effect is a later positivity from around 500–800 ms post-stimulus onset that is typically maximal over the left or central parietal scalp (Paller & Kutas, 1992; Rugg et al., 1995). The parietal effect is also referred to as the late positive component (LPC).

Numerous individual experiments have demonstrated associations between the mid-frontal ERP effect and familiarity, and between the parietal ERP effect and recollection. An early study by Rugg et al. (1998a) used a depth-of-processing manipulation to show a dissociation between mid-frontal and parietal old/new effects. The parietal old/new effect was elicited by deeply studied words (assumed to be recollected and familiar), but not shallowly studied words (assumed to be only familiar). This parietal effect differed topographically from an earlier mid-frontal effect associated with both types of words, suggesting distinct neural generators (see also Paller & Kutas, 1992; Rugg et al., 1998b). Other studies have used further manipulations known to differentially affect recollection and familiarity, such as matching versus mismatching items at study and test (Curran, 2000; Nessler et al., 2001), or manipulations of criterion placement (Azimian-Faridani & Wilding, 2006). Such approaches have contributed to the body of evidence supporting a dual-process account, although these experimental paradigms do not directly show that the different conditions they compare are associated with recollection and familiarity.

The strongest ERP evidence supporting a dual-process view comes from three experimental paradigms that use subjective or objective memory judgments to operationally define recollection and familiarity on a trial-by-trial basis. Each paradigm includes an experimental condition in which performance is assumed to be based on recollection – which may be accompanied by familiarity – and a condition in which performance is assumed to be based on familiarity without recollection. The logic of the recollection and familiarity contrasts is the same in all three paradigms: ERP effects specifically associated with recollection are computed by subtracting the ERP elicited by familiar items from those elicited by recollected items, and ERP effects specifically associated with familiarity are computed by subtracting the ERP elicited by a no-recognition condition from those elicited by familiar items. If recollection and familiarity are indeed based on distinct processes, a process-selective pattern should be observed, with a mid-frontal ERP effect for the familiarity contrast only, and a parietal ERP effect for the recollection contrast only.

In the Remember/Know (RK) paradigm, ERP effects are computed from three main experimental item types defined by subjective judgments of mnemonic experience. A studied item is classified as recollected if participants judge that they “Remember” (R) the context or details of their prior encounter with the item, and classified as familiar if participants just “Know” (K) that it was previously encountered but do not remember associated context or details (Gardiner, 1988; Tulving, 1985). A new, unstudied, item is classified as unrecognized if it is correctly rejected (CR). Alternatively, studied items attracting “new” judgments (misses) are sometimes used as the unrecognized condition (e.g., Duarte et al., 2004). The recollection-related ERP effect is computed by subtracting the ERP elicited by studied items attracting K judgments from that for R judgments, and the familiarity-related ERP effect is computed by subtracting the ERP elicited by the unrecognized items from the ERP elicited by studied items attracting K judgments (Düzel et al., 1997). A recent refinement of this paradigm recognizes a potential confound in these contrasts, which is that recollection is typically associated with high confidence while familiarity is associated with varying levels of confidence (Yonelinas, 1994). To address this possible confound, some studies have matched the level of confidence associated with recollected and familiar items in the recollection contrast by comparing ERPs for studied items judged Remembered with ERPs for studied items attracting only high-confidence K judgments (e.g., Woodruff et al., 2006). In other studies the contrast for the familiarity effect has been further refined, such that the critical test is whether the mid-frontal effect for items attracting K responses increases linearly across increasing levels of confidence (Yu & Rugg, 2010). A number of RK studies have found parietal ERP effects for recollection but not familiarity contrasts, accompanied by significant mid-frontal effects for familiarity but not recollection contrasts, supporting the dual-process prediction of a double dissociation (Woodruff et al., 2006; Yu & Rugg, 2010).

Unlike the Remember/Know paradigm, the source memory paradigm objectively tests whether retrieval judgments putatively based on recollection are correct or incorrect (Johnson et al., 1993). In a source memory task, participants study items such as words or pictures in two or more experimental contexts, such as the study modality or voice with which a word was spoken (Wilding et al., 1995; Wilding & Rugg, 1996). In the subsequent memory test, they are asked to identify which contextual feature was associated with each item at the time of encoding (e.g., a female or a male voice). Items associated with accurate source judgments (source hits) are assumed to be recollected, and may also be familiar, whereas items that are recognized as studied but for which source judgments are inaccurate (source misses) are assumed to be familiar primarily (Wilding et al., 1995), although they may sometimes be associated with retrieval of decision-irrelevant contextual information referred to as “non-criterial” recollection (Mulligan & Hirshman, 1997; Yovel & Paller, 2004). The recollection-related ERP effect is computed by subtracting the ERP elicited by source misses from the ERP elicited by source hits, separating source from item memory. The familiarity-related ERP effect is computed by subtracting the ERP elicited by correctly rejected unstudied items from the ERP for source misses. As in the RK paradigm, individual ERP studies using these contrasts have demonstrated double dissociations between the proposed ERP correlates of recollection and familiarity. The parietal effect has been elicited by recollection and the mid-frontal effect elicited by familiarity, the two occurring in distinct time windows and showing distinct scalp topographies (Curran & Hancock, 2007). Source memory tasks vary in some key details such as whether item recognition is assessed first or concurrently with the source judgment, and whether a “guess” option is included: the different versions offer varying precision in separating source from item memory (Batchelder & Riefer, 1990). Refinements of the source memory paradigm may also involve additional judgments of confidence, as in the refined RK paradigms (Diana et al., 2011; Woroch & Gonsalves, 2010). Woroch and Gonsalves (2010) used this approach to address the concern that if recollection, like familiarity, is graded, then ERPs that vary with confidence may potentially be driven by both processes.

In the associative recognition paradigm, recollection is operationalized in terms of the retrieval of associations between multiple (usually two) items. For example, participants are asked to study pairs of words, or names with faces. To measure memory for associations between items, the recognition memory test includes “same” pairs as well as “rearranged” pairs. Participants must judge whether or not items presented in the memory test are old or new, and if they are assessed to be old, whether they appear in the same pairing as at study (Donaldson & Rugg, 1998). The recollection condition is always correctly identified as same pairs (“associative hit”), and one of three alternative familiarity conditions may be used. The most frequent is correctly rejected rearranged pairs (associative CR), based on the assumption that recollection is more likely for same than rearranged pairs. For example, Donaldson and Rugg (1998) reported a larger parietal ERP old/new effect for associative hits than for associative CRs. The mid-frontal and the parietal ERP effects have been shown to dissociate temporally and topographically in associative memory tasks, as in the RK and source memory paradigms (e.g., Rhodes & Donaldson, 2007). However, it is recognized that correct rejection of rearranged pairs may not always be based on an absence of recollection because both items in the rearranged pairs were studied (although in a different combination). Rearranged pairs may therefore trigger recollection of one or more study episodes, allowing people to correctly reject them using a recall-to-reject strategy (Rotello & Heit, 2000). A better condition to identify familiarity without recollection is therefore the same pairs that are misclassified as rearranged (associative miss), because these pairs have been identified as studied but not recollected as “same.” Another option is to use rearranged pairs misclassified as same (associative false alarm or FA), although it is possible that such pairs attract inaccurate recollection (false or gist-based) rather than no recollection (Han et al., 2018; Kamp et al., 2016; Kriukova et al., 2013; Li et al., 2017, 2019; Rhodes & Donaldson, 2008).

There is a fourth experimental paradigm commonly used to study recollection and familiarity that we did not include in the present meta-analysis. In the recognition exclusion task, participants are asked to study items in two or more contexts as in a standard source memory task. At test, items from one source at a time are then designated as “targets,” while items from the other sources and unstudied items are both classified as “non-targets.” As in the associative memory paradigm, correct recognition of target items is thought to involve recollection of the original context, whereas correct rejection of non-target items may reflect the absence of target recollection, or the use of recall-to-reject strategies (Jacoby, 1991; Yonelinas & Jacoby, 2012). Based on such a rationale, some ERP studies have used exclusion tasks to investigate the control of recollection (e.g., Fraser et al., 2007; Herron & Rugg, 2003; Keating et al., 2017). However, the exclusion task offers only two response options in a single stage, unlike the source and associative recognition paradigms. This means that the exclusion paradigm provides no suitable condition to identify familiarity without recollection, because a non-target that is recollected and a non-target that is only familiar are both likely to be classified correctly as non-targets.

The evidence discussed thus far favors a dual-process view (Friedman & Johnson, 2000; Mecklinger, 2000; Rugg & Curran, 2007), but there are discrepant findings that substantiate single-process views. If a mnemonic strength-detection process does indeed support recognition memory, ERP effects associated with recollection and familiarity contrasts should be observed around the same time windows and scalp locations. In line with this notion, Yovel and Paller (2004) observed parietal ERP effects for both recollection and familiarity contrasts in a source memory task supplemented with judgments of non-criterial recollection, although the parietal maximum tended to be larger and longer lasting for recollection than familiarity. In a modified RK task with confidence judgments for abstract visual stimuli, Voss and Paller (2009) also reported a larger ERP magnitude for recollection than familiarity contrasts with indistinguishable central scalp distributions. These data are more consistent with a single-process view that the recollection contrast reflected stronger memory than the familiarity contrast.

### The current study

In this meta-analysis we asked to what degree the parietal effect and the mid-frontal effect are reliably associated with recollection and familiarity across studies. We conducted a systematic literature review and meta-analysis of recognition memory studies that measured mid-frontal and/or parietal ERP effects. Inclusion criteria encompassed studies that used the RK, source memory or associative memory paradigms described above (for details see *Methods – Inclusion criteria*), and measured mid-frontal and/or parietal ERP effects within a priori ranges of time-windows and scalp locations used in the literature.

If the dual-process account of recognition memory is indeed correct, we expected the parietal ERP effect to be larger for recollection than familiarity contrasts and the mid-frontal ERP effect to be larger for familiarity than recollection contrasts. We assumed that no ERP modulation is likely to be due to a single process, although the use of appropriate task contrasts can maximize process-purity (Luck, 2014; see *Discussion – Differences between experimental paradigms*). Nevertheless, process-purity can be improved by meta-analysis compared to individual studies if experiments employing multiple types of stimuli, test conditions, and participants are included. The above predictions were based on the common assumption that recollection and familiarity are independent processes, revisited in the *Discussion*. Such a crossover interaction would support a dual-process view that assumes the neural correlates of recollection and familiarity to be qualitatively distinct. Alternatively, if the single-process view is correct, we would expect a graded pattern in which one or both ERP effects are greater in magnitude for recollection (stronger memory) than for familiarity conditions (weaker memory), and greater for familiarity than when recognition is unsuccessful. A single dissociation in which the parietal effect is reliable for recollection contrasts but not familiarity contrasts while the mid-frontal effect is present at similar magnitude for both contrasts, or the other way around, would also be compatible with a single-process view.

A strength of a meta-analytic approach is that the first set of questions – whether the mid-frontal and parietal ERP effects are reliably found to be larger for recollection and for familiarity respectively – can be addressed by combining data from studies that only documented results for one ERP effect, and those that included only one relevant contrast. A limitation of a meta-analysis of published effect sizes is that it is not possible to test for interactions involving time and scalp topography, unlike in single experiments. We were able to address this limitation by performing these additional tests in a separate mega-analysis of six raw datasets.

## Methods

### Literature Search

**Fig. 1:**
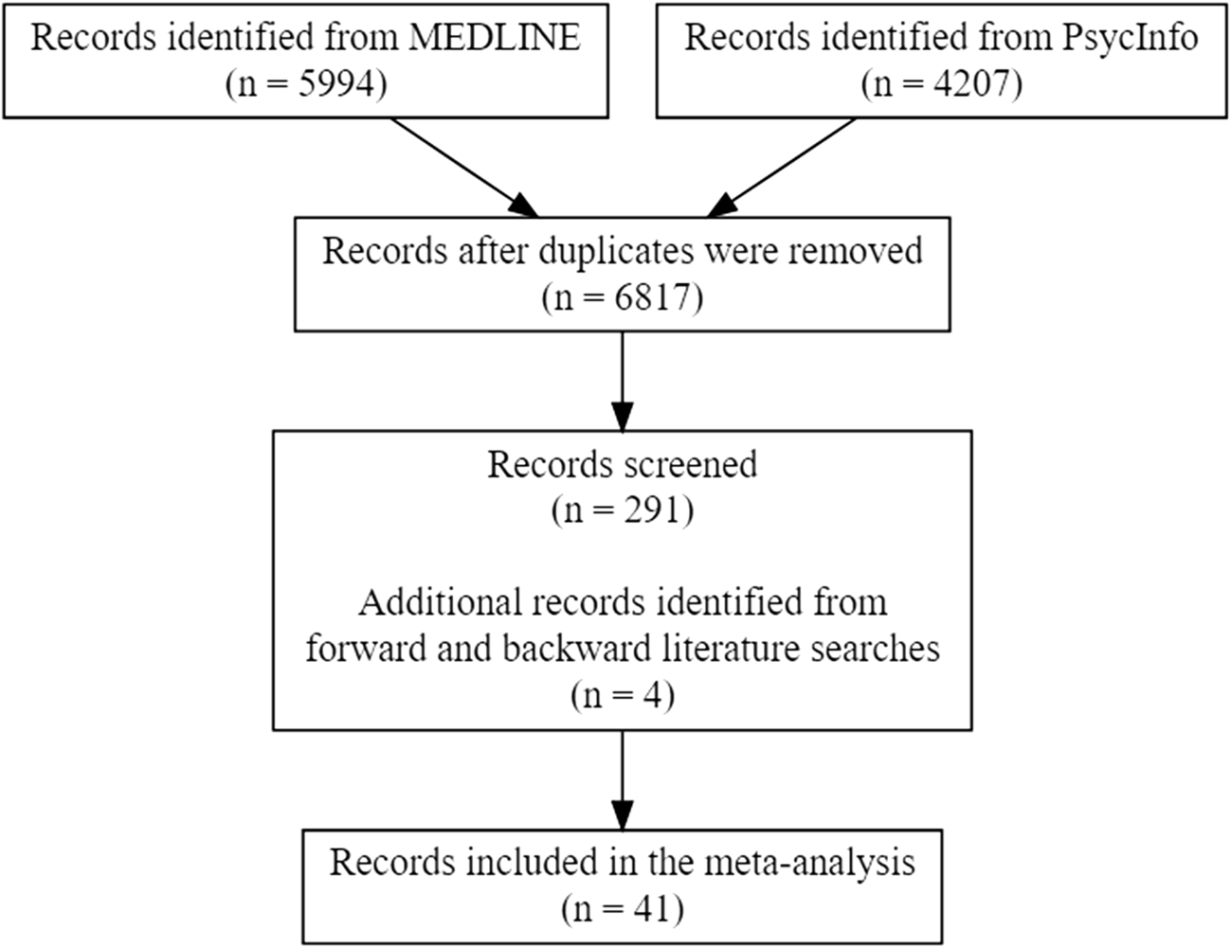
Diagrammatic representation of the stages of the systematic literature search. The full protocol, data, and code are available in the Online Supplemental Material and via the Open Science Framework at https://osf.io/acwtv/. The literature search yielded 6,817 articles (see Fig. 1 for details). We (SK, AM) first screened article titles and abstracts for relevance. If those fields were irrelevant to recognition memory and/or the event-related potentials of interest, we excluded the corresponding articles. We then read the full text of the remaining 291 articles to assess if they did indeed meet the inclusion criteria (see *Inclusion criteria* section). This inspection yielded 41 articles that could be included in the current meta-analysis

We searched for articles reporting ERP studies of recognition memory. The search was conducted in two main databases, MEDLINE and PsycINFO (via Ovid). The search terms used across both databases include recognition memory, recollection, familiarity, source memory, associative memory, and ERP (see Online Supplementary Materials (OSM) for the syntax). The literature search was initially conducted on April 2016 and updated on September 2018. Forward and backward literature searches were completed in July 2019. The search results are reported in Fig. 1, which follows the Preferred Reporting Items for Systematic Reviews and Meta-Analyses guidelines (PRISMA; Moher et al., 2009).

### Inclusion criteria

There were five sets of inclusion criteria. First, participants in the included studies must have been healthy, without any report of psychiatric or neurological conditions. They must have been between 18 and 35 years of age or, if the age of participants was not reported, drawn from an undergraduate population. Where other age groups or populations were also tested, these data were excluded.

Second, procedures in the studies must have involved the Remember-Know, source memory, or associative memory paradigms. Refined procedures involving graded response options such as confidence ratings were also included. The stimuli used must have been images or words. Images include faces, abstract symbols, and objects such as artifacts, animals, and people.

Third, EEG data must have been acquired during the retrieval phase of a recognition memory task, with stimulus-locked ERPs referenced to linked or average mastoids, and studies must have measured the mid-frontal and/or parietal old/new ERP effects (an exception to this was the six studies for which raw data were available, so mastoid-referenced ERPs could be computed even though the original papers used an average reference; see *Mid-frontal and parietal ERP effects by time window*). The mid-frontal effect must have been estimated at a central or symmetric combination of frontal electrodes including Fz, F1, F2, F3, F4, F5, F6, F7, F8, FCz, FC1, or FC2 (or the equivalent in a different electrode montage), in any time window between 250 and 600 ms post-stimulus onset. The parietal effect must have been estimated at a left-lateralized, central or symmetric combination Pz, P1, P2, P3, P4, P5, P6, CP1, CP2, CP3, CP4, CP5, or CP6 (or the equivalent in a different electrode montage), in any time window between 400 and 900 ms post-stimulus onset.

Fourth, contrasts used to estimate recollection ERP effects must have compared Remember Hit – Know Hit, Source Hit – Source Incorrect, Associative Same Hit – Associative Same Incorrect, Associative Same Hit – Associative Rearranged False Alarm (FA), Associative Same Hit – Associative Rearranged Correct Rejection (CR), or Remember – Confident Old. Contrasts used to estimate familiarity effects must have compared Know Hit – New CR, Source Incorrect – New CR, Associative Same Incorrect – New CR, Associative Rearranged FA – New CR, Associative Rearranged CR – New CR, or Confident Old – Confident New.

Fifth, statistical reports must have included effect sizes for at least one of the specified contrasts, or enough statistical information to calculate the effect size. Where reports lacked sufficient statistical information to calculate the effect size of interest in the current meta-analysis, we asked authors to provide sufficient statistical information or the data. If an article involved multiple experiments, only experiments that met the inclusion criteria were included.

### Data extraction and meta-analysis

The dependent measures in the current meta-analytic models were Hedge’s *g* effect sizes. To calculate Hedge’s *g*, we extracted the following statistical information from the articles: sample size, *F* or *t* values, degrees of freedom, or other effect sizes such as Cohen’s *d*. Conversion equations from Borestein et al., (2009) were used to convert this statistical information into *g*, variance of *g*, and standard error of *g*.

The meta-analysis was conducted in two stages. We first assessed each ERP effect separately for recollection and familiarity contrasts, then investigated differences in ERP effect sizes according to contrast. Mean weighted meta-effect sizes (g_w_) and 95% confidence intervals (CIs) were estimated with restricted maximum likelihood in the metafor package (version 2.1-0; (Viechtbauer, 2010) in R version 4.0.5 (R Core Team, 2022). Plots were generated with the ggplot2 package (version 3.3.5, Allen et al., 2019; Wickham, 2009). To accommodate variability of effect sizes between experiments, we included Study as a random effect. Studies with larger samples often report smaller variance of effect size and those variances were therefore weighted so that effect sizes with smaller variance influenced the overall meta-effect size more than those with larger variance (Hedges & Vevea, 1998). Significance tests for individual model coefficients (and the corresponding confidence intervals) were based on a standard normal distribution, and omnibus tests were based on a chi-squared distribution with degrees of freedom equal to the m (the number of coefficients tested). Tests for significant heterogeneity in the effect sizes (i.e., whether the variability in effect sizes is greater than that expected from sampling error alone) used the weighted least squares generalization of Cochran’s Q-test, which has a chi-squared distribution with k-1 degrees of freedom or (with moderators in the model), the QE-test with k-m-1 degrees of freedom (Lipsey & Wilson, 2001; Viechtbauer, 2010). Where multiple comparisons were required, we used a Holm-Bonferroni correction (Holm, 1979).

The first stage of the meta-analysis sought to establish whether the mid-frontal and parietal ERP effects for familiarity and recollection contrasts were each reliably present across studies. We therefore ran univariate meta-analytic models for each effect and contrast, using the rma.uni function in metafor. To check for outlier studies, we used quantile-quantile (Q-Q) plots, defining outliers using an interquartile range (IQR) rule of less than the lower quartile minus 1.5 × IQR or greater than the upper quartile plus 1.5 × IQR. Studies with undue influence on the analyses were also identified for exclusion if Cook’s distance was greater than 1 (Cook, 1977).

In the second stage, meta-regression models were constructed to compare multiple effect sizes per study in a linear mixed model framework (Baayen et al., 2008; Stram, 1996), using the rma.mv function in the metafor R package. To test whether each ERP effect was selective for its hypothesized process, models for the mid-frontal and parietal ERP effects included all studies that contributed effect sizes for both familiarity and recollection contrasts. The linear mixed models had the fixed effect factor of Process (Familiarity and Recollection), with Study as a random intercept and Process as a second random intercept nested within Study. To test for the hypothesized interaction between the two ERP effects according to memory process, a top-level model included all studies that contributed effect sizes for both ERP effects for both processes, and fixed effect factors of ERP effect (Mid-frontal and Parietal) and Process (Familiarity and Recollection). Study was modelled as a random intercept, with Contrast (four levels representing Effect × Process) as a second random intercept nested within Study. In all models, variance components were specified as “unstructured,” allowing both diagonals and off-diagonals of the variance-covariance matrix to be estimated.

### Bayesian meta-analysis

In addition to null hypothesis significance tests, we estimated equivalent Bayesian hierarchical linear models in the brms package (v2.16.3, Bürkner, 2017a, 2017b). Weakly informative prior distributions were set on the mean effect estimates and the cross-study heterogeneity tau (Gelman, 2006; Harrer et al., 2021; Williams et al., 2018). Priors on the effect sizes were unit normal distributions, and priors on cross-study heterogeneities tau were half-Cauchy with width = 0.5, corresponding to an expectation that the true heterogeneities would be positive and relatively substantial, given the differences in ERP measurement and analysis across experiments. For the second stage comparison of two ERP mid-frontal or parietal effect sizes per study, we specified Study as a random intercept with Process nested within it. Lastly, for the interaction model we modelled Study as a random intercept with Effect and Process nested within it. Posterior means (PMs) and 95% posterior credible intervals (PCIs) are also reported for each fixed effect. Heterogeneity estimates were not included in the Bayesian meta-analysis models (Vuorre, 2016). In the meta-analysis, where data were standardized effect sizes, we also derived the posterior probabilities that effects were greater than *g* = 0.2 if positive, or less than -0.2 if negative. 

### Effect size moderators

The literature suggests that a number of variables may modify the relations between mid-frontal and parietal ERP effect magnitude and familiarity versus recollection contrasts: (1) the experimental paradigm (i.e., RK, source memory, and associative memory paradigm), (2) the stimuli (verbal or pictorial), and (3) the relation between the stimuli (for source and associative paradigms, the possibility of unitization; Mayes et al., 2007). There were insufficient data points to test all of these in a joint model (see *Results*) so we address (2) and (3) in the *Discussion*. Regarding (1), we examined moderation by experimental paradigm for source memory versus RK studies.

### Publication bias

Potential publication biases are a concern for the interpretation of meta-analyses, if statistically significant findings are more likely to be published than null findings. Where bias is present, reported effects might be larger in studies with smaller samples in which effect size is overestimated. We addressed this form of publication bias by examining the asymmetry of funnel plots for each ERP effect and contrast, and performing two statistical tests: a random effects version of Egger’s test that regresses the effect sizes by sample sizes (Egger et al., 1997; Sterne & Egger, 2005), and a Begg’s rank correlation test, which evaluates the correlation between effect sizes and variances (Begg & Mazumdar, 1994).

### Mega-analysis of six datasets by time window

The mid-frontal ERP effect associated with familiarity is typically reported to be maximal around 300–500 ms post-stimulus, while the parietal ERP effect associated with recollection is maximal later around 500–800 ms (Rugg & Curran 2007). To test this a priori spatiotemporal prediction, data are required from both frontal and parietal scalp locations in both time windows.

No unpublished raw data were received, but raw data were available from six experiments for which the published ERPs had been computed using an average electrode reference (Curran & Hancock, 2007; Mollison & Curran, 2012, Experiments 1, 2, and 3; Ross et al., 2015; Strozak et al., 2016; see Table 1 for sample sizes). We were therefore able to conduct a direct within-participants mega-analysis as well as to extract effect sizes for the meta-analysis using an average mastoid reference, although as these raw data come from the same laboratory they reflect only a subset of the literature. These data were re-processed to obtain ERPs referenced to linked mastoids (see original papers for details of recordings). Preprocessing and ERP extraction were conducted in EEGLAB (version 13_5_4b) with ERPLAB (version 7.0.0) and MATLAB (R2015b) (for code see https://osf.io/acwtv/). First, gross artefacts were removed automatically using the Clean_rawdata EEGLAB function, with rejection criteria of 0.70 for channels, 20 for bursts and 0.3 for windows (v1.2), and a 0.1–40 Hz Hamming windowed-sinc FIR band-pass filter was applied, with a 50-Hz notch filter. Data were epoched to stimulus onset and further artefact rejection carried out using the Autoreject EEGLAB function with threshold of 200, probability threshold of 5 standard deviations (SD) and maximum of 5% rejected trials. Independent Components Analysis was then used to correct for ocular artefacts automatically using the ADJUST EEGLAB plugin (Mognon et al., 2011).

**Table 1:** List of included studies

Studies	Task	Stimuli	N	Mean age (y, range)	Effect size (standard error)			
					Frontal		Parietal	
					Familiarity	Recollection	Familiarity	Recollection
Addante et al. (2012)	Source	Words	25	NA	0.59 (0.08)	1 (0.14)	0.13 (0.02)	1.09 (0.15)
Bader et al. (2010) Definition condition	Associative	Words	20	23.65 (19-27)	0.94 (0.15)	0.04 (0.01)	0.79 (0.12)	0.23 (0.04)
Bader et al. (2010) Sentence condition	Associative	Words	20	23.8 (21-29)	1.05 (0.17)	-0.28 (0.04)	1.46 (0.23)	-0.07 (0.01)
Brezis et al. (2017)	RK	Words	23	NA (18-28)	NA	NA	NA	0.97 (0.14)
Cansino & Trejo-Morales (2008)	Source	Images	17	22.5 (NA)	1.44 (0.44)	2.4 (0.42)	-0.85 (0.4)	3.24 (0.45)
Cruse & Wildling (2009)	Source	Words	21	21 (18-27)	NA	NA	NA	1.7 (0.26)
Cruse & Wildling (2011)	Source	Words	16	21 (19-26)	0.17 (0.03)	1.45 (0.26)	1.54 (0.27)	1.2 (0.21)
Cruse (2010) Exp. 1	Source	Words	16	21 (18-29)	NA	NA	1.83 (0.32)	2.38 (0.42)
Cruse (2010) Exp. 3	Source	Words	21	22 (18-30)	NA	NA	NA	3.1 (0.48)
Cruse (2010) Exp. 4	Source	Words	16	21 (18-27)	NA	NA	2.17 (0.38)	1.33 (0.23)
Curran & Hancock (2007)	Source	Images	24	21 (18-27)	0.69 (0.1)	0.05 (0.01)	-0.39 (0.06)	0.71 (0.1)
De Chastelaine et al. (2009)	RK	Images	16	23 (21-25)	NA	1.29 (0.23)	NA	NA
Gao et al. (2015) Exp. 3	RK	Images	14	NA (19-25)	3.17 (0.6)	0.5 (0.09)	1.52 (0.29)	1.47 (0.28)
Gao et al. (2015) Exp. 4	RK	Images	15	NA (19-27)	1.93 (0.35)	1.12 (0.21)	2.33 (0.43)	1.59 (0.29)
Han et al. (2018)	Associative	Words	38	23.7 (NA)	NA	1.39 (0.23)	NA	1.36 (0.16)
Hou et al. (2013)	RK	Words	9	NA (19-25)	NA	1.51 (0.36)	NA	2.04 (0.48)
Kamp et al. (2016)	Associative	Words	38	23.33 (19-30)	NA	0.23 (0.03)	NA	0.34 (0.04)
Kriukova et al. (2013)	Associative	Words	14	23 (19-26)	NA	1.27 (0.24)	NA	0.98 (0.19)
Leynes & Crawford (2018)	Source	Images	28	NA (18-22)	0.04 (0.06)	0.14 (0.06)	0.03 (0.09)	0.09 (0.06)
Leynes & Mok (2017)	Source	Words	28	NA (18-22)	0.11 (0.05)	0.01 (0.02)	0.28 (0.1)	0.06 (0.04)
Leynes et al. (2017)	Source	Words	20	NA (18-22)	1.03 (0.14)	0.08 (0.01)	0.63 (0.09)	0.44 (0.06)
Li et al. (2016)	RK	Words	20	22 (NA)	0.89 (0.14)	-0.27 (0.04)	0.55 (0.09)	0.54 (0.09)
Li et al. (2017)	Associative	Words	16	22.31 (19-25)	0.99 (0.18)	NA	NA	4.13 (0.73)
Li et al. (2019)	Associative	Words	17	23.5 (20-26)	NA	0.7 (0.12)	NA	NA
Lucas et al. (2012) Exp. 1	RK	Words	20	20.6 (18-23)	NA	0.43 (0.07)	NA	0.41 (0.06)
Mao et al. (2015)	Source	Images	17	23.4 (NA)	NA	1.11 (0.19)	NA	0.81 (0.14)
Mollison & Curran (2012) Exp. 1	Source	Images	26	21.4 (18-28)	-0.12 (0.02)	0.93 (0.13)	-0.18 (0.02)	0.91 (0.13)
Mollison & Curran (2012) Exp. 2	Source	Images	28	21.2 (18-28)	0.9 (0.12)	0.01 (0)	0.21 (0.03)	0.65 (0.09)
Mollison & Curran (2012) Exp. 3	Source	Images	22	20.6 (18-29)	0.05 (0.01)	0.17 (0.03)	0.51 (0.08)	0.42 (0.06)
Rhodes & Donaldson (2008)	Associative	Words	22	21.9 (18-35)	1.22 (0.18)	3.32 (0.5)	NA	5.1 (0.77)
Ross et al. (2015)	Source	Words	60	20.7 (18-29)	0.04 (0)	0.09 (0.01)	0.28 (0.03)	0.23 (0.02)
Schaefer et al. (2011)	RK	Images	27	21 (NA)	0.97 (0.13)	0.6 (0.08)	0.22 (0.03)	0.4 (0.05)
Strozak et al. (2016)	RK	Words	24	21.46 (18-29)	0.3 (0.04)	-0.2 (0.03)	0.16 (0.02)	0.21 (0.03)
Tibon et al. (2014)	Associative	Images	33	23.3 (21-29)	NA	NA	NA	1.94 (0.24)
Trott et al. (1999)	RK	Words	16	NA (21-28)	1.45 (0.72)	0.57 (0.65)	-0.21 (1.93)	3.26 (1.42)
Vilberg et al. (2006)	RK	Images	15	NA (18-24)	1.15 (0.21)	NA	NA	1.4 (0.25)
Wang et al. (2012)	RK	Words	23	21 (NA)	2.3 (0.34)	0.27 (0.04)	1.67 (0.25)	2.44 (0.36)
Wang, Mao, Li, Lu et al. (2016a)	Associative	Words	20	22 (NA)	1.61 (0.25)	NA	NA	2.89 (0.46)
Wang, Mao, Li, Wang et al. (2016b)	Associative	Words	16	22.4 (19-25)	2.1 (0.37)	NA	NA	1.47 (0.26)
Wilding & Rugg (1996) Exp. 1	Source	Words	16	NA	0.74 (0.13)	0.72 (0.13)	2.49 (0.44)	0.25 (0.04)
Wilding & Rugg (1996) Exp. 2	Source	Words	16	NA	0.43 (0.08)	0.45 (0.08)	0.7 (0.12)	1.5 (0.26)
Wilding (1999)	Source	Words	17	20 (18-26)	0.9 (0.15)	2.04 (0.35)	0.59 (0.1)	1.65 (0.28)
Wolk et al. (2006)	RK	Words	15	NA (18-22)	2.03 (0.37)	NA	NA	2.06 (0.38)
Woodruff et al. (2006)	RK	Words	16	NA (18-27)	0.98 (0.17)	0.24 (0.04)	0.91 (0.16)	2.06 (0.36)
Woollams et al. (2008)	RK	Words	15	NA	NA	NA	NA	1.28 (0.23)
Woroch & Gonsalves (2010)	Source	Images	21	NA (18-25)	NA	NA	NA	1.1 (0.17)
Yu & Rugg (2010)	RK	Images	23	19.4 (18-22)	1.85 (0.27)	0.25 (0.04)	0.53 (0.08)	3.35 (0.49)

*Note*. Memory tasks used were Remember/Know (RK), source memory, and associative memory. The term NA refers to information that was not reported in the original study

In order to determine whether the results were comparable with those of the meta-analysis, we again initially analyzed individual ERP effects and proceeded to a direct test of the interaction between ERP effect and contrast. Mean ERP old-new effects for familiarity and recollection conditions were computed across all candidate electrode locations for the frontal effect, and across all candidate left-sided electrode locations for the parietal effect (see *Inclusion criteria*). For statistical analysis, we constructed linear mixed effects models in R using the lme4 function (version 1.1-18.1) with participant grand-average ERP old-new effects as the dependent measure. Fixed effects factors were Scalp Location (Frontal and Parietal), Time Window (Early and Late), Process (Familiarity and Recollection), and Experiment (1–6). Fixed effects factors were mean-centered and scaled, and the Experiment factor was coded using orthogonal polynomial contrasts. For Bayesian inference, we fitted corresponding models using the brms package with 4 MCMC chains each with 1,000 warmup and 9,000 post-warmup draws. Priors on the fixed effects were unit normal distributions, and priors on the standard deviations were half-Cauchy with width = 0.5. We computed the posterior probabilities that effects were greater than 0 if positive, or less than 0 if negative (except for the multiple contrasts for the effects of Study). In both classical and Bayesian models, participants were modelled as a random intercept.

## Results

### Meta-analysis

The 41 included studies collectively reported 47 experiments that contributed to the meta-analysis summarized in Table 1. Across all experiments, 1,000 participants (597 female, 362 male, 41 not specified, mean age range 18.7–26.4 years) were included.

### Mid-frontal and parietal ERP effects for familiarity and recollection

**Fig. 2:**
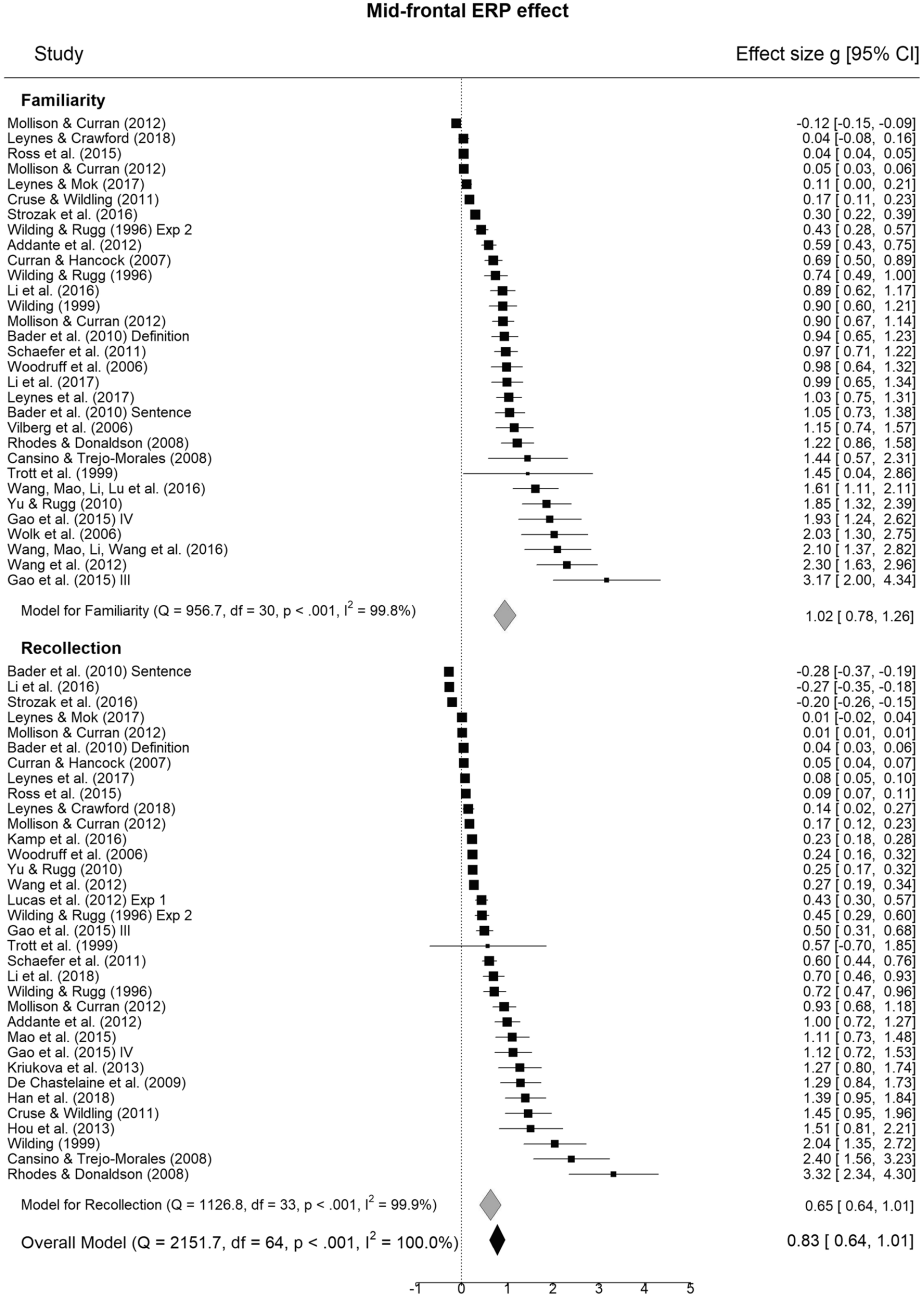

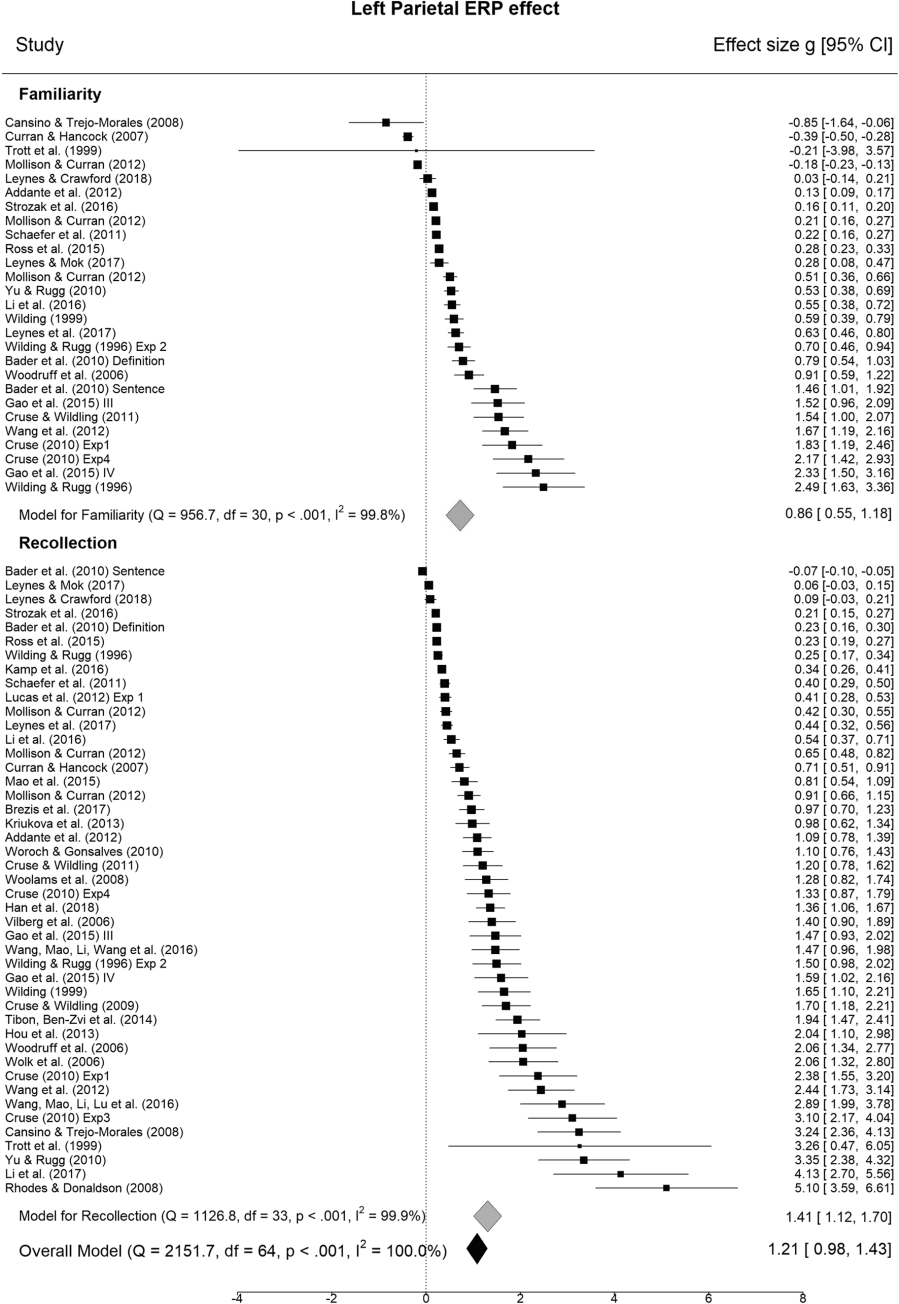
Forest plots for mid-frontal and parietal event-related potential (ERP) effects. Individual study values (filled squares) represent standardized mean differences (effect sizes) for ERP effects. The overall meta-analytic effect size across all contrasts is given below (filled black diamond) with model statistics. Subgroup model statistics and meta-analytic effect sizes are plotted separately for familiarity and recollection contrast subgroups. Error bars represent 95% confidence intervals. For definitions of quantities see *Methods – Meta-analysis*

We first sought to test reliability of the two ERP effects for each mnemonic process. Meta-analysis of individual effects revealed robust mid-frontal and parietal ERP effects for both familiarity and recollection (for forest plots see Fig. 2; p-values corrected over four tests). The mid-frontal effect was large for familiarity (31 studies; *g* = 0.94, 95% CI = 0.70–1.19, *z* = 7.56, *p* < .001; PM = 0.93, PCI = 0.67–1.19, *p*_>0.2_ = 1.00), with substantial heterogeneity over studies (*Q*(30) = 956.71, *p* < .001). The effect was also robust and moderate in size for recollection (34 studies; *g* = 0.63, 95% CI = 0.39–0.86, *z* = 5.20, *p* < .001; PM = 0.62, PCI = 0.39–0.87, *p*_>0.2_ = 1.00), but again with substantial heterogeneity (*Q*(33) = 1126.84, *p* < .001). The parietal ERP effect size was large for recollection (45 studies; *g* = 1.30, 95% CI = 1.01–1.60, *z* = 8.68, *p* < .001; PM = 1.27, PCI = 0.97–1.57, *p*_>0.2_ =1.00) and also for familiarity (27 studies; *g* = 0.72, 95% CI = 0.42–1.02, z = 4.71, *p* < .001; PM = 0.70, PCI = 0.39–1.00, *p*_>0.2_ = 1.00), with high heterogeneity over studies (*Q*(44) = 1524.85, *p* < .001; *Q*(26) = 725.90, *p* < .001). Q-Q plots and IQR tests did not reveal any outliers for any of the ERP effects or contrasts, and no studies showed undue influence (Cooks Distance < 1 in all cases; maximum = 0.33). However, as a check of the robustness of results we re-ran the principal analyses of differences in ERP effects according to contrast after excluding one study (Trott et al., 1999) that had large variances, particularly for the left parietal effect. Excluding this study made no substantive difference to the results.

### Differences in ERP effects for recollection and familiarity

The next set of analyses sought to test two key hypotheses stemming from the dual-process account: the mid-frontal ERP effect size will be greater for familiarity than recollection and the parietal ERP effect size will be, in contrast, greater for recollection than familiarity. Linear mixed model meta-regression that compared the mid-frontal effect sizes for recollection verses familiarity did not reveal a significant difference (k = 26 studies, g = -0.31, *z* = -1.41, *p* = .157). The corresponding Bayesian model provided some evidence for a difference (PM = -0.31, PCI = -0.66–0.05; *p*_<-0.2_ = .73). The model for the parietal effect also did not reveal a significant difference in effect size between recollection and familiarity (k = 27 studies, g = 0.45, *z* = 1.74, *p* = .082), and there was relatively weak Bayesian evidence for a difference (PM = 0.29, PCI = -0.14–0.72, *p*_>0.2_ = .66). In both cases there was substantial cross-study heterogeneity (*QE*(50) = 72669.36, *p* < .001, and *QE*(52) = 10936.34, *p* < .001). The differences in both ERP effects according to contrast remained non-significant when the study with very large variances (Trott et al., 1999; Fig. 2) was removed from the analysis. There was therefore inconclusive evidence for process-selectivity of the mid-frontal and parietal ERPs when analyzed separately.

**Fig. 3:**
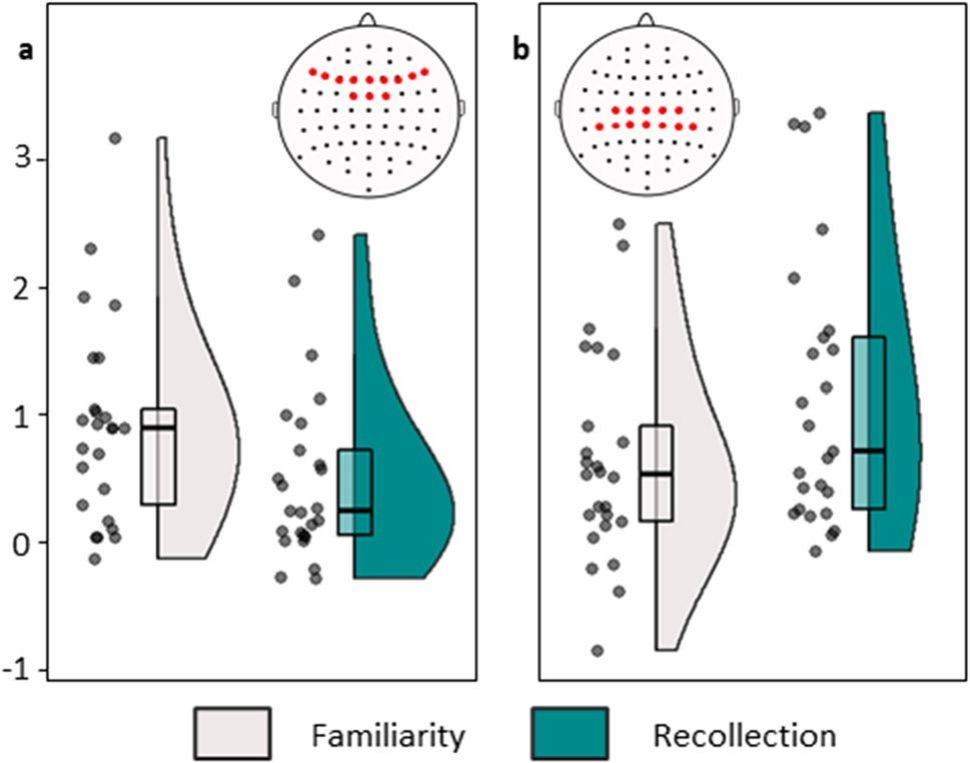
Interaction plot of effect sizes by event-related potential (ERP) effect and contrast. (**a**) Mid-frontal ERP effect. (**b**) Parietal effect. Filled circles show effect sizes (Hedge’s g) for each study, shaded areas show the probability density function of the data, and boxplots show the median and interquartile range. Insets illustrate the electrode inclusion criteria for each effect (note that mid-frontal effects had to have been measured symmetrically, whereas electrode groupings for parietal effects could have been left-sided or symmetrical; see *Methods – Inclusion criteria*)

Direct analysis comparing process-selectivity between the two ERP effects included the 25 studies with estimates of all four effect sizes in a final, joint meta-regression model. This analysis revealed a significant interaction between ERP effect (mid-frontal and parietal) and Process (recollection versus familiarity) (beta = 0.74, *z* = 2.82, *p* = .005), with strong Bayesian evidence for the interaction (PM = 0.64, PCI = 0.18-1.10, *p*_>0.2_ = .97). There was again substantial residual heterogeneity in the model (*QE*(96) = 3116.39, *p* < .001). The interaction effect remained significant when the study with very large variances (Trott et al., 1999) was removed from the analysis (24 studies; beta = 0.69, *z* = 2.26, *p* = .008; PM = 0.62, PCI = 0.15–1.08, *p*_>0.2_ = .97; see Fig. 3).

### Moderator effects

The following analysis sought to test whether the observed interaction between ERP effect and mnemonic processes was moderated by experimental paradigm (remember/know, source memory, and associative memory). Data were available from 16 studies that had used an RK task (12 studies with a standard RK task and four that also included confidence judgments), 20 that had used a source memory task, and 11 that had used an associative memory task. In order to examine possible moderator effects by experimental paradigm, the standard and variant RK studies were grouped together. Associative memory studies were excluded due to insufficient numbers except for analysis of the parietal effect for recollection, for which there were ten such studies (there were six for the mid-frontal effect for familiarity, seven for the mid-frontal effect for recollection, and two for the parietal effect for familiarity). A total of 23 studies provided data for both ERP effects for both contrasts (nine studies for RK, 14 for source memory).

**Fig. 4:**
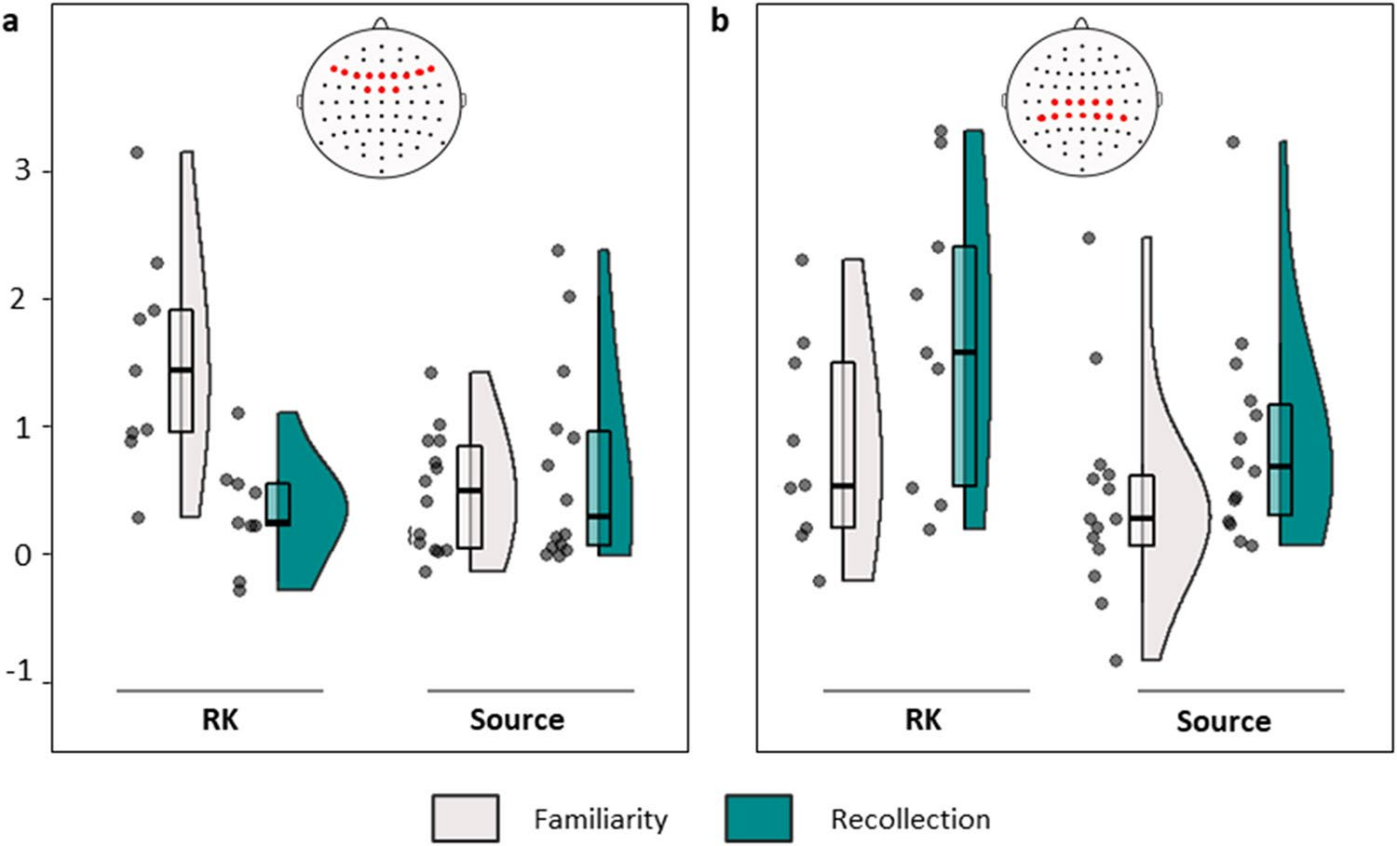
Interaction plot of effect sizes by experimental paradigm and process for each event-related potential (ERP) effect. (**a**) Mid-frontal ERP effect. (**b**) Parietal effect. Filled circles show effect sizes (Hedge’s g) for each study, shaded areas show the probability density function of the data, and boxplots show the median and interquartile range. Insets illustrate the electrode inclusion criteria for each effect (see *Methods – Inclusion criteria*)

The mid-frontal effect for familiarity (but not recollection) appears larger for RK than for source studies (Fig. 4; see OSM Figs. 1 and 2 for corresponding forest plots). Separate tests of the moderation by experimental paradigm of each ERP effect and contrast revealed the mid-frontal effect for familiarity to be significantly larger in RK than source memory studies, with strong Bayesian evidence for an appreciable moderator effect (see Fig. 4; p-values corrected over four tests) (*QM*(1) = 16.38, *p* < .001; PM = 0.85, PCI = 0.36–1.35, *p*_>0.2_ = 1.00), with no difference for recollection (*QM*(1) = 0.37, *p* = .531; PM = 0.26, PCI =-0.24–0.76, *p*_>0.2_ = .96). The size of the parietal effect did not differ significantly by paradigm for either recollection or familiarity (*QM*(1) = 1.24, *p* = .266, *QM*(1) = 0.935, *p* = .334) and Bayesian evidence for a moderator effect was minimal in both cases (PM = 0.45, PCI = -0.27–1.17, *p*_>0.2_ = .24; PM = 0.29, PCI = -0.37–0.94, *p*_>0.2_ = .39). Substantial residual heterogeneity was observed in the mid-frontal effect models for familiarity and recollection (*QE*(23) = 559.24 and *QE*(25) = 838.61; p < .001) and in the corresponding parietal effect models (*QE*(23) = 637.68; p < .001, and *QE*(33) = 636.78).

In post hoc tests we assessed differences by paradigm and process for each ERP effect in the 23 studies for which this was possible (p-values corrected over two tests). For the mid-frontal ERP effect, a linear mixed model with moderating factors of paradigm and contrast revealed a significant overall effect of moderators (*QM*(3) = 26.66, *p* < .001) with a highly significant interaction of paradigm and process (beta = 1.36, *z* = 4.28, *p* < .001) and strong Bayesian evidence for a moderator effect (PM = 1.03, PCI = 0.44–1.61, *p*_>0.2_ = 1.00). This showed that the moderation by paradigm differed for familiarity and recollection as the separate analyses suggested. For the parietal effect, although a similar model showed an overall effect of the moderators (*QM*(3) = 8.59, *p* = .035), the interaction was not significant (beta = 0.53, *z* = 0.93, *p* = .354; PM = 0.19, PCI = -0.63–1.04, *p*_>0.2_ = .49). Finally, the higher-order analysis examined moderation by experimental paradigm of the interaction between ERP effect and process. The impact of task paradigm differed strongly between the mid-frontal and parietal effects as shown by a significant overall moderator effect (*QM*(7) = 43.6, *p* < .001) and three-way interaction of paradigm, ERP effect, and process (beta = 1.96, *z* = 3.21, *p* = .0001; PM = 1.09, PCI = 0.29–1.87, *p*_>0.2_ = 1.00).

## Publication bias

**Fig. 5:**
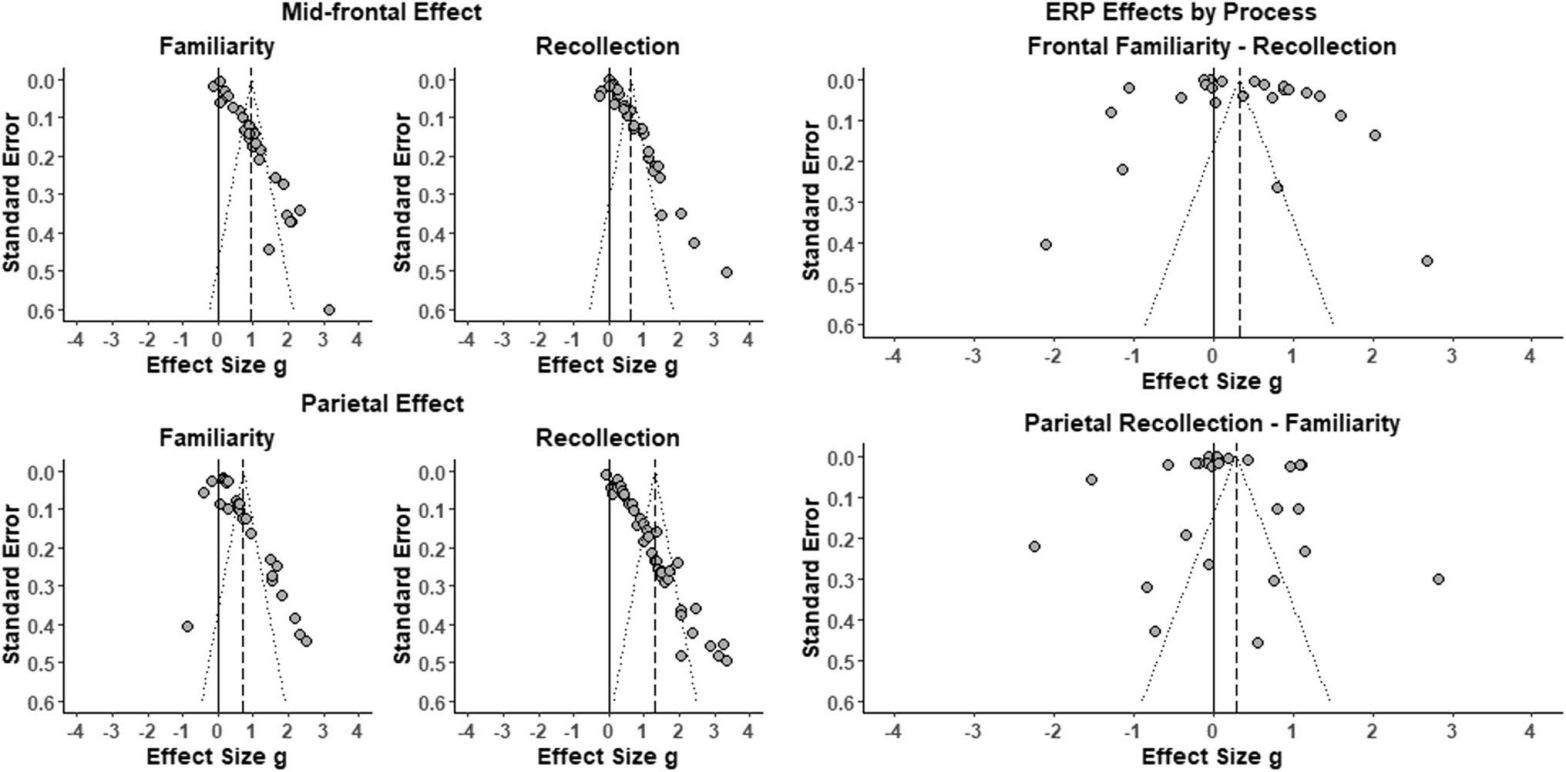
Funnel plots of event-related potential (ERP) effect sizes. The group of four plots on the left shows the relationships between effect size (Hedge’s g) and its standard error for mid-frontal and parietal effects for recollection and familiarity processes. The pair of plots on the right shows this relationship for the differences in each ERP effect according to process

Inspection of the funnel plots for each ERP effect and contrast of interest (Fig. 5) suggested publication bias where studies with larger effects tend to have lower precision. This notion was substantiated by significant Egger’s tests (for the mid-frontal effect for familiarity, *z* = 15.20, for recollection, *z* = 12.85; for the left parietal effect for familiarity, *z* = 4.03*;* for recollection, *z* = 22.31, *p* < .001 for all, corrected over four tests). A correlation between effect sizes and variances was also present for the parietal effect (for recollection, tau = 0.37, p < .001; for familiarity, tau = 0.37, p = .007), but not for the mid-frontal effect (for familiarity, tau = 0.13, p = .327; for recollection, tau = 0.13, p = .275). Analogous analysis of publication bias was conducted for process-selectivity in the two ERP effects (Fig. 5). This set of analyses for the differences in each ERP’s effect sizes according to process revealed no significant publication bias (for mid-frontal effect, *z* = -0.10 *p* = .484; for parietal effect, *z* = 0.09, *p* = .508, corrected over two tests).

### Mega-analysis of six datasets by time window

**Fig. 6:**
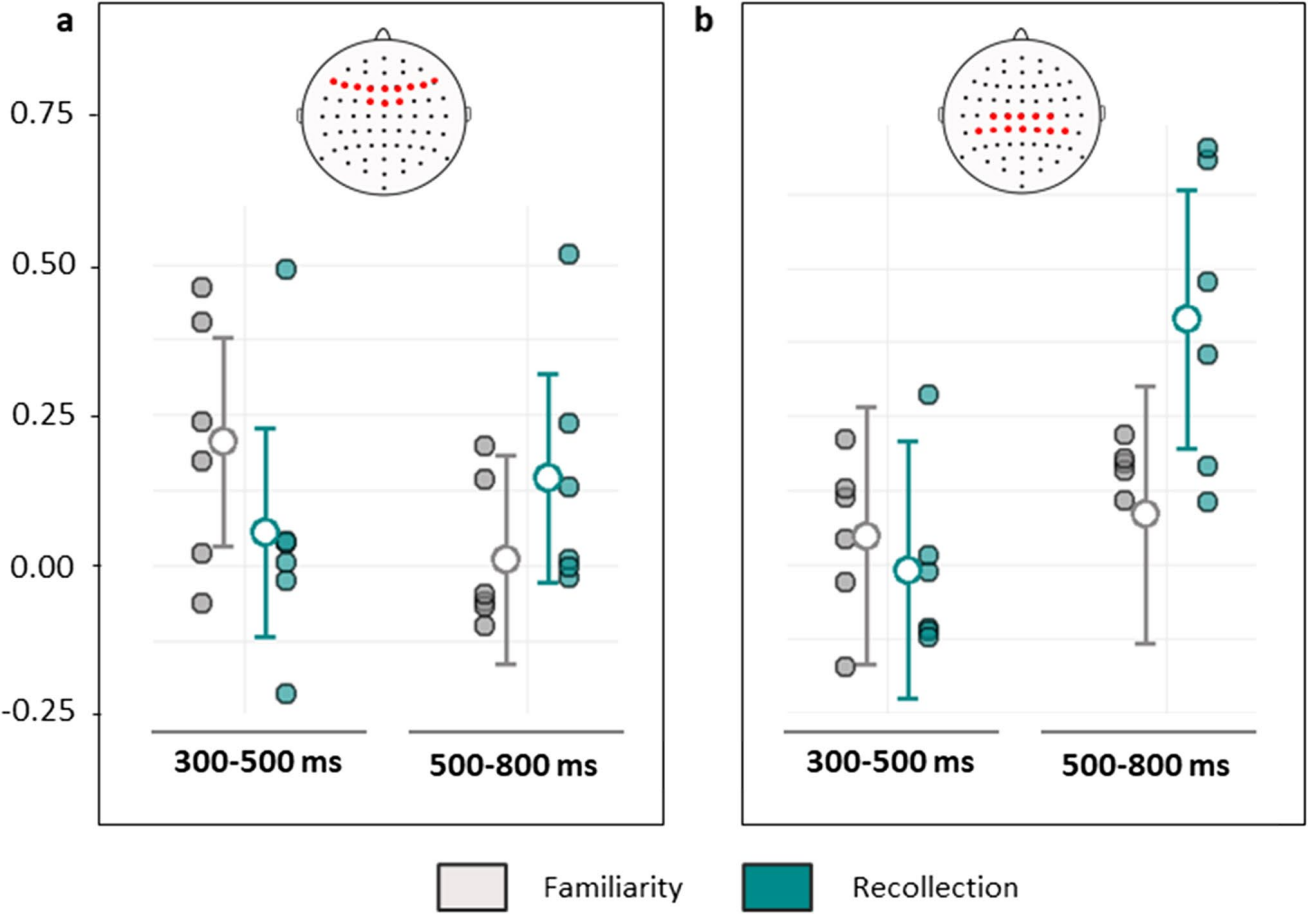
Interaction plots of effect sizes in the mega-analysis by time window and process for each event-related potential (ERP) effect. (**a**) Mid-frontal effect. (**b**) Parietal effect. Filled circles show marginal means for each experiment, and bars show the median and interquartile range across experiments. Insets illustrate the electrode inclusion criteria for each effect (see *Methods – Inclusion criteria*)

To test the dual-process theory predictions that the mid-frontal and parietal ERP effects will differ in their timings and scalp locations, we analyzed raw EEG data from six experiments (Curran & Hancock, 2007; Mollison & Curran, 2012, Experiments 1, 2, and 3; Ross et al., 2015; Strozak et al., 2016; see Fig. 6). We constructed two linear mixed model with factors of Process (familiarity and recollection), Time Window (300–500 and 500–800 ms) and Experiment (1–6) to test the predictions that the mid-frontal effect would be larger in the earlier time window for familiarity than recollection, and that the parietal effect would be larger in the later time window for recollection than familiarity. For each ERP effect, the dual-process account predicted an interaction between Process and Time Window across studies. For the mid-frontal effect, the results were consistent with this prediction, revealing an interaction between process and time window (*F*(1, 543) = 10.51, p < .001, MSE = 3.42; PM = 0.07, PCI = 0.03–0.11, p_>0_ = 1.00) but no main effect of process (*F*(1, 543) = 0.03, p = .86, MSE = 0.01; PM = 0.00, PCI = -0.04–0.05, p_>0_ = .57). Post hoc tests by time window showed the predicted larger mid-frontal ERP for familiarity than recollection in the early time window (*F*(1,181) = 6.10, p = .029, MSE = 1.90; PM = 0.07, PCI = 0.02–0.13, p_>0_ = 0.99). In the late window the mid-frontal effect was, unexpectedly, significantly larger for recollection than familiarity (*F*(1,181) = 4.51, p = .035, MSE = 1.53; PM = 0.07, PCI = 0.00–0.13, p_>0_ = .98; see Fig. 6).

For the parietal effect, the effect of process also depended on time window as predicted by a dual-process account, demonstrated by a significant two-way interaction between these factors (*F*(1, 543) = 12.22, p = .001, MSE = 6.32; PM = 0.10, PCI = 0.04–0.15, p_>0_ = 1.00) and a main effect of process (*F*(1, 543) = 6.04, p = .014, MSE = 3.13; PM = 0.07, PCI = 0.01–0.12, p_>0_ = .77). Post hoc analysis revealed a larger parietal effect for recollection than familiarity contrasts across the six experiments in the late time window only (*F*(1, 181) = 18.61, p < .001, MSE = 9.17, PM = 0.16, PCI = 0.09–0.24, p_>0_ = 1.00; for the early window, *F*(1,181) = 0.51, p = .475, MSE = 0.28, PM = 0.03, PCI = -0.05–0.11, p_>0_ = .99). Nevertheless, the modulation of process effects by time window for the parietal ERP also varied across the six experiments, as shown by a significant two-way interaction of process and experiment (*F*(1, 543) = 6.04, p = .014, MSE =3.13) and a three-way interaction (*F*(5, 543) = 2.48, p = .031, MSE =1.28). Post hoc tests by experiment revealed that the difference in process-selectivity between time windows was driven mainly by two studies, with significant interactions of time window with process in Curran and Hancock (2007) (*F*(1, 69) = 14.13, p < .001, MSE = 8.00; PM = 0.29, PCI = 0.13–0.44, p_>0_ = 1.00) and Mollison and Curran (2012) Experiment 1 (*F*(1, 69) = 6.30, p = .014, MSE = 2.79; PM = 0.16, PCI = 0.03–0.29, p_>0_ = .90). This interaction effect was not significant in Ross et al. (2015) (*F*(1, 69) = 1.06, p = .30, MSE = 0.52; PM = 0.05, PCI =-0.05–0.14 , p_>0_ = .84), Strozak et al. (2016) (*F*(1, 69) = 0.45, p = .50, MSE = 0.52; PM = 0.07, PCI = -0.14–0.29, p_>0_ = .74), or in Mollison and Curran’s Experiment 2 (*F*(1, 69) = 0.00, p = .96, MSE = 0.00; PM = 0.00, PCI =-0.11–0.11, p_>0_ = .48) or Experiment 3 (*F*(1, 69) = 0.014, p = .91, MSE = 0.00; PM = 0.00, PCI = -0.07–0.08, p_>0_ = .46).

While the preceding analysis showed the two ERP effects differed by mnemonic processes in the hypothesized time window (although effects were variable across studies), a stronger test for a dissociation is whether the observed temporal patterns of responses to familiarity and recollection contrasts also vary with scalp location. To test this prediction, we ran a linear mixed model with the additional factor of Scalp Location (mid-frontal and parietal). A significant two-way interaction of process and time window across locations confirmed the marked temporal dissociation described above (*F*(1, 1267) = 15.93, p < .001, MSE = 6.72, PM = 0.08, PCI = 0.05– 0.12, p_>0_ = 1.00). Contrary to the dual-process theory prediction of topographic differences between processes which should be more pronounced in the relevant time windows, this effect was not modified significantly by scalp location (*F*(1, 1267) = 0.525, p = .469, MSE = 0.22, PM = 0.01, PCI = -0.02–0.05, p_>0_ = .77). This top-level model also revealed a four-way interaction with experiment where temporal characteristics of the parietal effect differed significantly among the six studies, while those for the mid-frontal effect did not (*F*(5, 1267) = 2.59, p = .024, MSE = 1.09; for parietal, *F*(5,543) = 2.48, p = .031; for mid-frontal, *F*(5,543) = 1.35, p = .241).

## Discussion

The present meta-analysis sought to determine whether a double dissociation of the ERP correlates of recollection and familiarity is reliable over studies, as predicted by dual-process theories of recognition memory. The mid-frontal ERP effect was expected to be selective for familiarity, i.e., larger for familiarity than recollection contrasts, and the parietal ERP effect to be selective for recollection, i.e., larger for recollection than familiarity contrasts (at least if the two processes are independent). The alternative, single-process view predicted that both ERPs would be larger for stronger than weaker memories. In that case, both effects should be present for both recollection and familiarity contrasts since both compare stronger with weaker memory conditions. The effect of process on the two ERPs would therefore be indistinguishable. Meta-analyses for each ERP effect and process revealed significant mid-frontal and parietal effects for both familiarity and recollection contrasts, confirming that the ERP effects were reliably related to memory over studies. In line with dual-process theory, direct comparisons revealed a strong interaction between effect and process, but although each ERP effect differed according to mnemonic process in the direction predicted, these differences were not independently significant. A direct comparison of the two ERP measures by time window in the mega-analysis further revealed that each ERP was selective for its predicted process only in the expected time window. These findings show that, at least in these six studies, familiarity and recollection were temporally dissociable, showing the pattern predicted by the dual-process view.

The complementary process-selectivity of the mid-frontal and parietal ERP effects is incompatible with the single-process view that familiarity and recollection differ merely in the strength of the mnemonic signal (Squire et al., 2007; Yovel & Paller, 2004). The single-process prediction of equivalent ERP differences for recollection compared to familiarity and familiarity compared to unrecognized items assumes that ERPs scale linearly with memory strength. Under a weaker assumption of monotonicity, one ERP contrast might be significant and the other not, but both ERPs should follow the same pattern, and the crossover pattern we observe would not be observed.

Despite this overall pattern of results, the data offer at best partial support for the subsidiary dual-process theory predictions that each ERP effect would be selective for its respective process (Rugg et al., 1998b; Rugg & Curran, 2007). In the subset of six studies the scalp locations of the two ERPs also did not differ. Another potential challenge for the dual-process view is that meta-analytic effect sizes for both ERP effects were reliably greater than zero for both processes. This may in part have reflected lack of process-purity of experimental contrasts (discussed below). Dual-process accounts can also accommodate a small mid-frontal ERP in recollection contrasts because, like single-process accounts, they assume that familiarity is graded and can under some conditions contribute to “recollection” responses. The large effect size for the parietal ERP for familiarity contrasts is more problematic, since dual-process theories characteristically assume that recollection occurs for some items but fails for others in a threshold process (Onyper et al., 2010; Yonelinas, 1994; Yonelinas et al., 2010). Dual-process theories nevertheless predict a double dissociation between two process-selective ERP effects, and so are supported, albeit weakly, by the overall findings of the current meta-analysis. There are also other potential explanations for the finding of a significant parietal effect for “familiarity” contrasts, even if the parietal effect indeed reflects a distinct recollection process that differs from familiarity. We discuss these in the next section.

### Differences between experimental paradigms

Clearer results for the mid-frontal ERP effect emerged from exploratory analysis of the moderating effects of experimental paradigm. This analysis was motivated by concerns about the process-purity of experimental contrasts designed to isolate familiarity and recollection. Although ERPs can in principle distinguish between predictions of dual- and single-process theories of recognition memory, evidence might be obscured by contamination of estimates of familiarity by recollection, and the other way around, in ways that depend on the experimental task (Allan & Rugg, 1998). We found that effect sizes for the two ERPs depended on memory process only in studies using the RK paradigm, as opposed to the source memory paradigm. In RK studies, the mid-frontal ERP effect was significantly larger for familiarity than recollection contrasts, while parietal ERP effect sizes were non-significantly larger for recollection contrasts in both RK and source memory paradigms. Indeed, in the source memory studies neither difference was significant. Although relatively few studies contributed data to this analysis (16 for RK and 20 for source memory), evidence for a paradigm-dependent modulation of the mid-frontal effect was strong, pointing to stronger support for dual-process models from studies using the RK than the source paradigm. This difference likely reflects a well-recognized limitation of the source memory paradigm used in recognition memory research: many of the studies included in this meta-analysis operationalized familiarity as the absence of recollection on source-incorrect trials. The source-incorrect condition may be contaminated by trials on which the item as well as the source was forgotten, attenuating the neural correlates of familiarity, particularly if the overall level performance is low (Mollison & Curran, 2012). A better way to separate item and source memory is to use a two-stage recognition procedure in which participants first indicate whether they recognize the item probe, and then judge its source, although participants may find it difficult to make separate judgments (Migo et al., 2012). Of the two studies included here that used such a two-stage procedure, one reported the largest familiarity-related mid-frontal ERP of the source memory studies (Cansino & Trejo-Morales, 2008) and the other a moderate sized effect (Wilding & Rugg, 1996) (see OSM Fig. 1).

It is also likely that the source-incorrect trials involved retrieval of decision-irrelevant (non-criterial) episodic information. While source tasks (unlike RK tasks) yield an objective measure of contextual recollection by requiring participants to make judgments about a specific feature of the study episode, non-criterial recollection of the item or of other aspects of the study context is typically not measured. Insofar as participants recollect non-criterial study information on source-incorrect trials, the familiarity contrasts tend to be contaminated with recollection, so the parietal ERP effect will be present for familiarity as well as recollection contrasts. This confound may impact neural correlates of both processes and may mask process-selectivity even if the dual-process theory is correct (Allan et al., 1998; reviewed by Migo et al., 2012). The parietal recollection ERP is also likely to be attenuated because (for example) when source hits are compared with source misses, both of these response types might involve at least some degree of recollection.

To address this concern, source tasks can be refined by adding a confidence or RK judgment to assess non-criterial as well as source recollection (Yonelinas & Jacoby, 1996). This approach was adopted in 11 of the 20 included studies, although these studies either analyzed ERPs according to RK responses and source memory responses separately, or collapsed data across high and low confidence levels, for example to obtain sufficient trials for ERP analysis (Addante et al., 2012; Cruse, 2010; Cruse & Wilding, 2009, 2011; Leynes et al., 2017; Leynes & Crawford, 2018; Leynes & Mok, 2017; Mao et al., 2015; Mollison & Curran, 2012; Woroch & Gonsalves, 2010). Similar limitations apply to the associative memory paradigm, although there were not enough such studies in the present meta-analysis to formally examine differences from the other two paradigms (two experiments contributed all four ERP effect sizes; Bader et al., 2010). A further potential confound in a source task is the possibility that familiarity can support accurate source judgments due to unitization, as discussed below.

Although the original RK procedure avoids the problem of non-criterial recollection, it is prone to a different concern, at least if memory strength and memory process are confounded (Squire et al., 2007). In the original procedure, despite instructions, Know judgments may be given when participants experience weak familiarity and Remember judgments when they experience strong familiarity, as well as when they experience recollection. This confound will particularly tend to attenuate neural correlates of familiarity, such as the mid-frontal ERP (although we did not observe this pattern). More generally, if memory process and memory strength are confounded, different neural correlates associated with the experiences of recollection and familiarity may reflect cognitive operations linked to the level of confidence rather than the mnemonic signal (Henson et al., 1999, 2000). These issues can be circumvented by adding confidence judgments to the RK task (Migo et al., 2012; see *Introduction*). Qualitatively, the data from the four (of 16) current RK studies using the latter approach suggest that a stronger double dissociation of ERP correlates of recollection and familiarity is obtained using this approach, although there are too few studies for a formal statistical comparison (Hou et al., 2013; Wang et al., 2012; Woodruff et al., 2006; Yu & Rugg, 2010). This refined RK approach may nevertheless be imperfect if, for example, people set a criterion for recollection judgments (if recollection is graded), so weak recollection might accompany K judgments leading to contamination of their neural correlates.

A further limitation of the included associative recognition experiments concerns the contrast between associative hits and associative correct rejections that was used to identify recollection (Wang, Mao, Li, Lu, et al., 2016; Y. Wang, Mao, Li, Wang, et al., 2016). As noted in the *Introduction*, associative correct rejections are not the ideal familiarity condition because participants may use a recall-to-reject strategy. Just as for non-criterial recollection, this contamination of the familiarity contrast with recollection means that the “familiarity” contrasts will tend to elicit neural correlates of recollection and the “recollection” contrasts will show attenuated effects. This concern can be avoided by using associative miss trials as a familiarity condition, if there are sufficient trials. As noted in the *Methods*, associative false alarms – recombined pairs that are incorrectly identified as studied – or a combination of associative misses and false alarms – can also be used as a familiarity condition, but with the risk of confounding false recollection with familiarity.

### Other potential moderators of ERP effects

We originally planned to examine two further potential moderators of the mid-frontal and parietal ERP effects, but in neither case were there sufficient studies for formal meta-analysis. The first of these moderators is the potential for unitization of stimuli as a single compound item at encoding, something that might have obscured the process-selectivity of the parietal ERP effect (Mayes et al., 2007). Examples include two same-modality items such as words in associative tasks, or items and item-bound source features such as color (for a review, see Yonelinas et al., 2010). Evidence from ROC (Diana et al., 2008) and neuropsychological data (Diana et al., 2010; Quamme et al., 2007) suggests that familiarity can support associative and source memory for such unitized conjunctions, although it has been argued that the same ROC findings can also be explained by a some-or-none graded recollection process (Onyper et al., 2010). If unitized stimuli are used to define source or associative hits as recollection conditions in ERP studies but rememberers can correctly identify these items based on familiarity, the neural correlates of recollection are likely to be weaker. In such studies, differences in the parietal ERP according to process might therefore be obscured even if a dual-process account is correct (Ecker et al., 2007; Migo et al., 2012; Mollison & Curran, 2012; Nyhus & Curran, 2009; Rhodes & Donaldson, 2007, 2008). Our meta-analytic findings are compatible with this interpretation. While the results for source memory tasks taken alone most parsimoniously suggest a single underlying mnemonic process, the fact that the parietal ERP was significantly larger for recollection than familiarity contrasts in RK but not in source memory tasks (Fig. 4) is more compatible with the view that process-purity was compromised by unitization in source tasks. That said, the between-paradigm difference here was mainly driven by greater process-selectivity of the mid-frontal rather than the parietal ERP. Potential reasons for this latter finding have been addressed above.

The second potential moderator of interest is the meaningfulness of the stimuli. In some of the included studies, a mid-frontal ERP effect was observed only when meaningful stimuli such as nameable objects were used, leading to the proposal that this ERP reflects conceptual fluency rather than familiarity (Paller et al., 2007; Voss & Paller, 2009; Yovel & Paller, 2004). An alternative view is that conceptual fluency and familiarity rely on overlapping processes (see Wang & Yonelinas, 2012). The stimulus-dependency or otherwise of the mid-frontal effect is therefore a critical issue for adjudicating between dual- and single-process theories, because it offers an alternative explanation for the prosed neural correlate of familiarity.

### Assumptions about the relationship between familiarity and recollection

Dual-process models of recognition memory have made three main assumptions about the relationship between familiarity and recollection processes: exclusivity, redundancy, or independence (for review, see Yonelinas et al., 2002). The first assumption, that recognition can be based on one or other process but not both (Gardiner, 1988), predicts the existence of a strong double dissociation between the neural correlates of familiarity and recollection, such that the two correlates do not co-exist. However, it has been clear for some time that the weight of the evidence is incompatible with this assumption (Skinner & Fernandes, 2007).

A second possibility is that familiarity and recollection are redundant, i.e., all recollected items are also familiar but only a subset of familiar items are (also) recollected (Joordens & Merikle, 1993). The parietal ERP should therefore be clearly identified by recollection contrasts that (as here) subtract familiarity-related activity from recollection-related activity. Recollection contrasts might reveal a small mid-frontal effect if familiarity strength is greater for recollected than familiar-only items, but they should not reveal an effect if the strengths are equivalent. Moreover, any mid-frontal effect identified by a recollection contrast should be smaller than that elicited by familiarity contrasts that subtract activity elicited by unrecognized items from familiarity-related activity. The mid-frontal effect might also be expected to be more pronounced in RK than in source and associative tasks. This is because familiarity is operationalized directly in terms of Know responses in the RK task but, as was discussed above, is inferred in source and associative tasks when recollection of a criterial feature of the study episode fails, leaving room for the influence of non-criterial recollection. Moreover, as the instruction in RK tasks is to respond Know only when items are familiar *and* not recollected (Yonelinas & Jacoby, 1995), the parietal effect should be absent or small for familiarity contrasts under the redundancy assumption. While our data do not support the latter prediction, the overall double dissociation between the two neural correlates is consistent with redundancy. The specific finding that the mid-frontal ERP was more selective for familiarity in RK than source tasks also fits well with a redundancy rather than an independence model.

A common alternative assumption about the relationship between recollection and familiarity is that the two processes demonstrate stochastic independence. That is, items can be familiar only, recollected only, or both familiar and recollected ( Jacoby et al., 1993; Mandler, 1980). Unlike redundancy, independence predicts a parietal effect to be present for familiarity contrasts if familiar items are also at least weakly recollected (on the assumption that recollection is graded). A unique further prediction derived from an independence assumption is that, in the RK paradigm, average familiarity strength should be greater for items judged as familiar (Know) than for those judged as recollected (Remember). This prediction follows because although recollected and unrecollected items will be familiar to some degree, making a Know judgment depends upon an above-criterion familiarity signal, whereas familiarity can be weak or absent for items judged Remembered. Thus, under the independence assumption only, the mid-frontal effect is predicted to be smaller for Remembered than for Known items. We did not observe such a pattern here, and are aware of only one electrophysiological study, using magnetoencephalography (MEG), that reported this pattern of findings (Evans & Wilding, 2012; see also Johnson et al., 2013). The lack of support for this prediction could be due to a lack of sensitivity to detect what might often be a quite subtle effect, and this unique prediction could be tested more directly in future studies. Although the picture is complicated by the lack of process purity described above, the present results tend to favor the redundancy over the independence assumption.

### Timing of ERP recognition memory effects

A key piece of evidence that can bolster the inference that two ERP effects represent distinct underlying mnemonic processes is the finding that they occur at different times (Luck, 2005; Rugg & Curran, 2007; Rugg et al., 1998a). Moreover, dual-process theories of recognition memory make a prediction about temporal order of the two processes, with familiarity being fast and automatic while recollection is slower and controlled (Mandler, 1980). Testing for a temporal dissociation between the mid-frontal and parietal ERP effects requires measures derived from multiple time windows for both effects, which were not provided by the majority of studies in the current meta-analysis. Analysis of such temporal data for six experiments revealed the predicted temporal sequence, with both ERP effects showing process-selectivity that was maximal in the expected time window. The unexpected finding that the mid-frontal effect was also significantly larger for recollection than familiarity contrasts in the later time window suggests that recollection-related ERP effects extend beyond parietal scalp locations.

Two ERP effects that reflect distinct processes and underlying neural generators should also show different spatial distributions over the scalp. No such topographic dissociation was observed across these six studies, although individual experiments have reported such patterns (see Friedman & Johnson, 2000; Rugg & Curran, 2007). Topographic differences are difficult to detect because of the poor spatial resolution of the scalp EEG, and it is likely that ERP effects reflect overlapping activity from multiple deep as well as superficial brain sources. Even functional magnetic resonance imaging (fMRI), with its superior anatomical resolution, has so far revealed mixed evidence for the proposal that recollection depends on the hippocampus and familiarity on the surrounding medial temporal cortex (Eichenbaum et al., 2007). In parallel with the neuropsychological data, evidence associating recollection specifically with the hippocampus is stronger than evidence associating familiarity with the medial temporal cortex (Eichenbaum et al., 2007). Some of the same issues have also arisen as discussed above for ERP studies, for example the possibility of a confound between memory strength and memory process (see Migo et al., 2012; Wais et al., 2008). One way forward may be to optimize temporal and spatial resolution by trial-by-trial EEG-fMRI fusion (Hoppstädter et al., 2015), although to our knowledge such studies have not yet used specific contrasts to isolate familiarity and recollection-related activity.

### Limitations

Although the current results favor a dual-process rather than a single-process view of recognition memory, the support is relatively weak. Despite strong evidence for the interaction between memory process and ERP effects overall, there was inconclusive evidence that the two ERP effects taken separately each showed the predicted difference for familiarity versus recollection. Effect sizes were also markedly heterogenous in each case, suggesting that much of the across-study variance in the magnitude of the two ERP effects remains to be explained. We have already considered some ways that this heterogeneity may be reduced in future studies by improving task design. Without improvements in task design, however, sample sizes will need to be larger to reliably detect differences in the mid-frontal and parietal ERPs according to process, given the effect sizes we find here. Samples of at least 66 will be needed for one-tailed dependent *t*-tests to achieve 0.8 power to detect the modulation of the mid-frontal effect (*g* = -0.31), and at least 32 for the modulation of the parietal effect (*g* = 0.45), compared to a sample size range in the current meta-analysis of nine to 60 (Table 1).

A related problem is the observed publication bias for the two ERP effects when analyzed separately for each process: studies involving small numbers of participants tended to report larger effect sizes with lower precision. This bias might reflect a “file drawer problem”, where statistically null results have been less likely to be published than those that are statistically significant (Rosenthal, 1979). Such problems often skew meta-analyses to yield false positive results (Borenstein et al., 2009). Although publication bias is always of concern, our main aim here was to use the degree of process-selectivity in these two ERP effects to clarify evidence for dual- versus single-process theories of recognition memory. Unlike for the separate measures of ERP effects for each process, equivalent tests did not reveal evidence of bias in these further analyses. These findings suggest the observed publication bias is toward significant ERP findings in general, as opposed to those supportive of either a dual- or a single-process view.

Considering the keen interest in neural correlates of recognition memory as a means to test dual- versus single-process views, it is surprising that our systematic literature search yielded only 47 experiments from which we were able to extract effect sizes. We had to exclude experiments if they did not report sufficient statistical results. Some statistical reports were, for example, abbreviated to include only p-values without the corresponding *F-* or *t-* statistics. Many reports did not include descriptive statistics in either tables or plots for the time-windows of interest. For these reasons, we were unable to estimate the size of reported effects, and such studies had to be excluded from the current meta-analysis. Even if studies reported comprehensive statistical details, they were nevertheless excluded if the analysis approach used did not yield sufficient information to derive the required effect sizes for our research question. For example, some studies collapsed the analysis across factors such as hemisphere, time window, and response condition, obscuring the specific measures needed to evaluate mid-frontal and parietal ERP effects for familiarity and recollection contrasts. We also excluded studies that compared ERPs elicited by recollected items with those for correctly rejected new items, rather than with familiar items. Again, such an approach is perfectly adequate for addressing other research questions, although in some cases (as noted by the authors on enquiry) there were also insufficient trials in a familiarity condition. Differences in data preprocessing can also make it difficult to integrate results over studies if, for example, ERP magnitudes cannot be compared across different reference electrodes, and raw data were not available in most such cases.

Substantial effort is being made in the ERP community to establish agreement on recording, processing, analysis, and reporting standards (e.g., Keil et al., 2014; Luck, 2014; Picton et al., 2000). Data sharing is, however, still relatively uncommon. For the current meta-analysis, several authors dug into archives to extract further information so that older studies could be included. Other data were lost for reasons including use of outdated storage devices such as floppy discs and compact discs. A particularly useful exception was the availability of the raw data for the six studies that enabled us to reanalyze and include them even though the original papers had used an average electrode reference. Moreover, these data allowed us to refine our tests of dual-process theory assumptions with a mega-analysis. To our knowledge, this is the first study to report evidence from direct analysis of raw data across multiple ERP studies of recognition memory, as well as the first to conduct a meta-analysis of such studies (although see Donoghue & Voytek, 2022, for a novel, text-mining based approach). It is now widely acknowledged that sharing raw data in publicly accessible repositories allows future researchers to conduct analyses not envisaged by the original authors, and to test novel research questions (Foster & Deardorff, 2017). This has already been applied in at least one large-scale mega-analysis of EEG event-related responses in other cognitive domains (Bigdely-Shamlo et al., 2020). Furthermore, data may be shared for studies with unpredicted or null results, reducing bias (Wilkinson et al., 2016). The potential benefits of data sharing are particularly pronounced for large-scale multidimensional data like EEG, that no individual study addressing a single research question can come close to fully characterizing (Markiewicz et al., 2021). This endeavor will be encouraged by establishing and implementing common data guidelines, such as the Brain Imaging Data Structure (Gorgolewski et al., 2016), as well as by researcher incentives (American Society for Cell Biology, 2012).

### Conclusions

The present study revealed ERP effects associated with familiarity and recollection to be distinguishable both in terms of their process-selectivity and their temporal sequence, despite their unclear selectivity when taken alone. Together, the data offer qualified support for dual- compared to single-process theories of recognition memory but point to a need for further refinement of experimental ERP approaches. Adjudication between these two theoretical views will also be facilitated by opportunities for mega-analysis of shared raw data from a larger, representative set of studies.

### Supplementary Information

ESM 1 (DOCX 1103 kb)
