# Supplementary material for: A meta-analysis of event-related potential correlates of recognition ﻿memory
Source: Psychon Bull Rev. 2023 Jul 11;30(6):2083–105. doi: 10.3758/s13423-023-02309-y (PMC10728276; doi:10.3758/s13423-023-02309-y)
Supplement: Supplementary file 1 — (DOCX 1103 kb) [file 13423_2023_2309_MOESM1_ESM.docx]

# Supplementary Materials

Table 1

*Literature Search Syntax Used in MEDLINE.*

| ID | Description | Syntax |
| --- | --- | --- |
| 1 | Memory related index terms | memory, episodic/ or memory, long-term/ or Mental Recall/ or "recognition (Psychology)"/ or "retention (Psychology)"/ or verbal learning/ or paired-associate learning/ or association learning/ |
| 2 | Memory related command terms | (source memory or associative memory or item memory or recognition memory or episodic or recollect*).ti,ab. |
| 3 | Memory AND familiar* | (familiar* and memory).ti,ab. |
| 4 | Memory related syntax | 1 OR 2 OR 3 |
| 5 | ERP related index terms | Evoked Potentials/ or Electroencephalography/ |
| 6 | ERP related command terms | (erp* or event-related potential* or 'event related potential*' or evoked-potential* or evoked potential*).ti,ab. |
| 7 | ERP related syntax | 5 OR 6 |
| 8 | Combining memory and ERP terms | 4 AND 7 |
| 9 | Recognition index term | "Recognition (Psychology)"/ |
| 10 | ERP AND recognition index term | 7 AND 9 |
| 11 | Associative memory index term | Association Learning/ |
| 12 | ERP AND associative memory index term | 7 AND 11 |

Table 2

*Literature Search Syntax Used in PsycInfo.*

| ID | Description | Syntax |
| --- | --- | --- |
| 1 | Memory related index terms | memory/ or autobiographical memory/ or episodic memory/ or explicit memory/ or false memory/ or implicit memory/ or long term memory/ or verbal memory/ or visual memory/ or "recall (learning)"/ or source monitoring/ or "recognition (learning)"/ |
| 2 | Memory related command terms | (source memory or associative memory or item memory or recognition memory or episodic or recollect*).ti,ab. |
| 3 | Memory AND familiar* | (familiar* and memory).ti,ab. |
| 4 | Memory related syntax | 1 OR 2 OR 3 |
| 5 | ERP related index terms | evoked potentials/ or electrical activity/ or electroencephalography/ or electrophysiology/ or encephalography/ or exp Electroencephalography/ or exp Evoked Potentials/ |
| 6 | ERP related command terms | (erp* or event-related potential* or 'event related potential*' or evoked-potential* or evoked potential*).ti,ab. |
| 7 | ERP related syntax | 5 OR 6 |
| 8 | Combining memory and ERP terms | 4 AND 7 |
| 9 | Recognition memory index term | "recognition (learning)"/ |
| 10 | ERP AND recognition index term | 7 AND 9 |
| 11 | Associative memory index term | "recall (learning)"/ |
| 12 | ERP AND associative memory index term | 7 AND 11 |


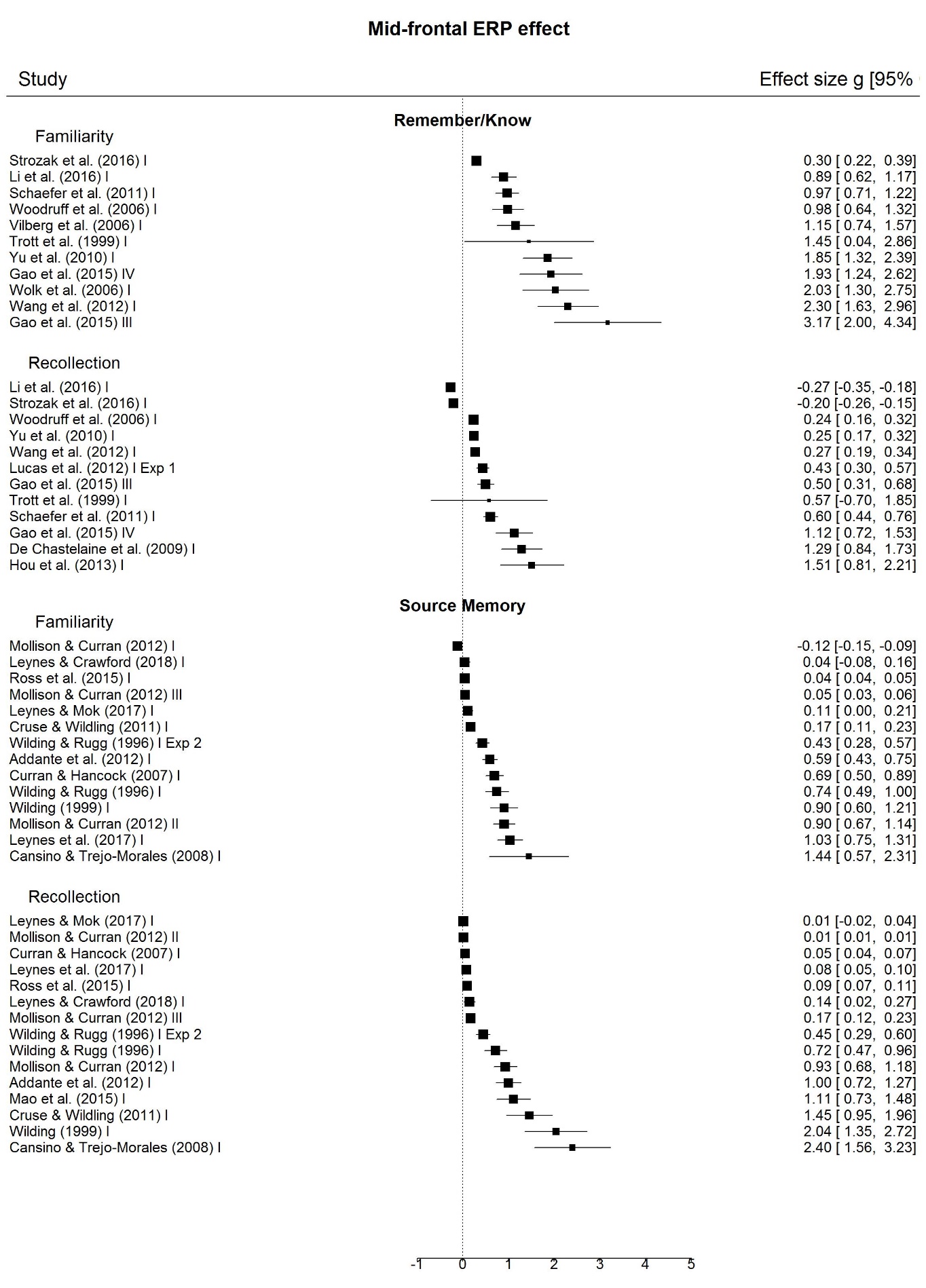

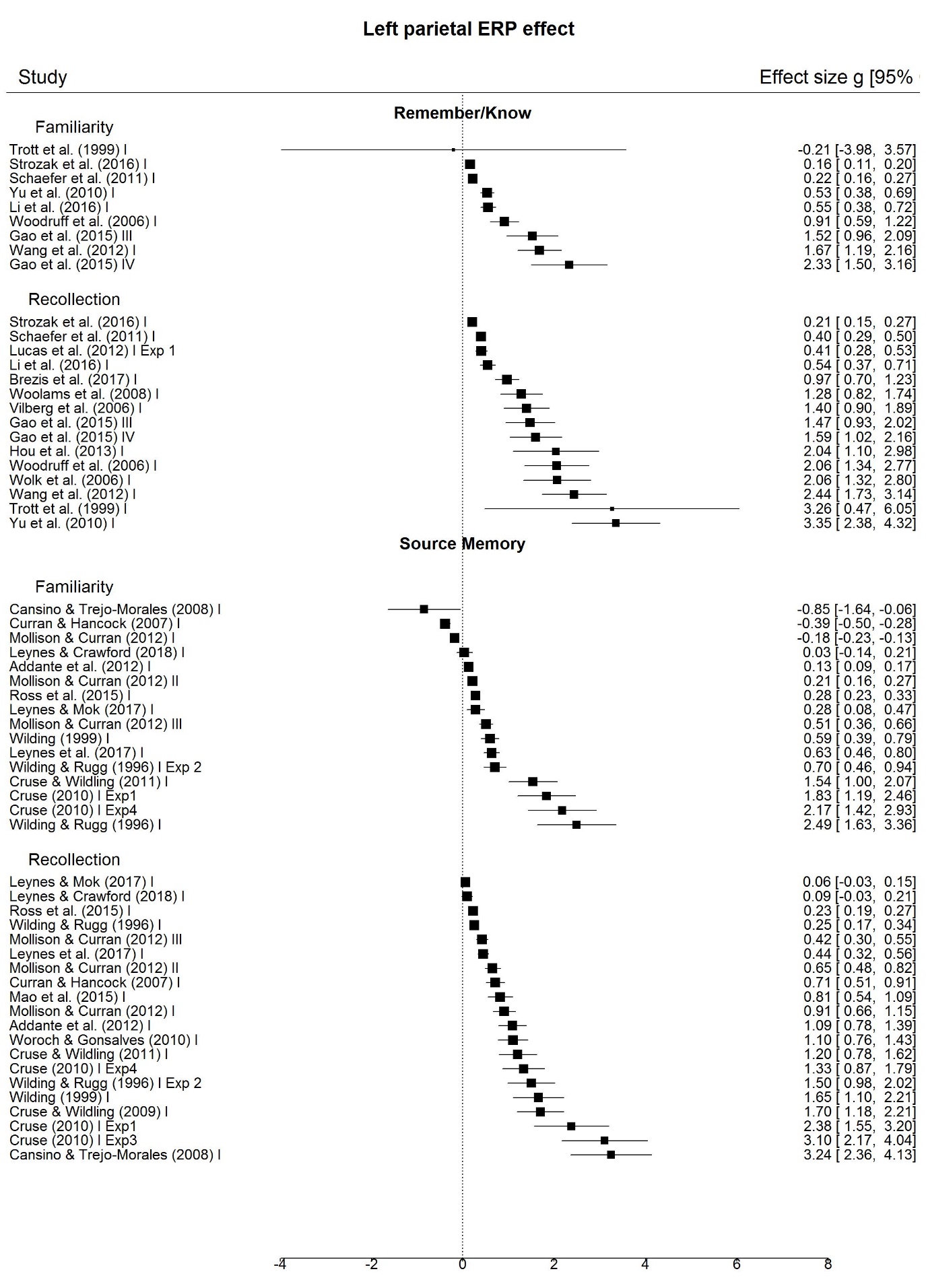


Supplementary Figure 1. Forest plots for mid-frontal and parietal ERP effects by experimental paradigm. Individual study values (filled squares) represent standardized mean differences (effect sizes) for ERP effects. Error bars represent 95% confidence intervals. For definitions of quantities see Methods, Meta-analysis.
